# Radiotracer stereochemistry affects substrate affinity and kinetics for improved imaging of system x_C_^-^ in tumors

**DOI:** 10.7150/thno.63237

**Published:** 2022-01-24

**Authors:** Hannah E. Greenwood, Richard Edwards, Norman Koglin, Mathias Berndt, Friedrich Baark, Jana Kim, George Firth, Eman Khalil, Andre Mueller, Timothy H. Witney

**Affiliations:** 1 School of Biomedical Engineering & Imaging Sciences, King’s College London, London, UK; 2 Life Molecular Imaging GmbH, Berlin, Germany

**Keywords:** [^18^F]FRPG, positron emission tomography, xCT, system x_C_^-^, cancer imaging

## Abstract

Amino acid utilization is perturbed in cancer cells, which rewire their metabolism to support cell survival and proliferation. This metabolic reprogramming can be exploited for diagnostic purposes through positron emission tomography imaging of fluorine-18 labeled amino acids. Despite its promise, little is known regarding transporter-recognition of non-natural amino acid stereoisomers or their utility for cancer imaging. We report here the synthesis and *in vivo* characterization of a radiolabeled amino acid (*R*)-4-(3-^18^F-fluoropropyl)-L-glutamate ([^18^F]FRPG) and compared its tumor imaging properties to the 4*S*-isomer, [^18^F]FSPG.

**Methods:**

[^18^F]FRPG and [^18^F]FSPG uptake was assessed in H460 lung cancer cells, with efflux measured 30 min after removal of exogenous activity. Specificity of [^18^F]FRPG for system x_C_^-^ was further examined following transporter inhibition and blocking studies with system x_C_^-^ substrates. [^18^F]FRPG and [^18^F]FSPG pharmacokinetics was next quantified in mice bearing subcutaneous A549, H460, VCAP and PC3 tumors, with mice bearing A549 tumors imaged by PET/CT. To better-understand differential tumor retention, radiometabolite analysis was performed on tissue and blood samples after imaging. Next, [^18^F]FRPG and [^18^F]FSPG retention in lipopolysaccharide-treated lungs were compared to an orthotopic H460 lung cancer model. Finally, the sensitivity of [^18^F]FRPG to manipulation of the redox environment was examined in cell and *in vivo* models.

**Results:**

[^18^F]FRPG was specifically transported across the plasma membrane by the cystine/glutamate antiporter system x_C_^-^ and retained at high levels in multiple tumor models. Conversely, [^18^F]FRPG was rapidly extracted from the blood and cleared from tissues with low system x_C_^-^ expression. Due to its favorable imaging properties, tumor-to-blood ratios ≥10 were achieved with [^18^F]FRPG, which were either equal to or greater than [^18^F]FSPG. In addition, [^18^F]FRPG retention in orthotopic lung tumors with high system x_C_^-^ expression was 2.5-fold higher than inflamed tissue, allowing for clear tumor visualization. *In vivo*, [^18^F]FRPG and [^18^F]FSPG were metabolized to a single species, with [^18^F]FRPG showing a higher percentage of parent radiotracer in tumors compared to [^18^F]FSPG. [^18^F]FRPG was sensitive to redox manipulations and tumor retention was reduced following treatment with liposomal doxorubicin in mice bearing ovarian tumors.

**Conclusions:**

Given the fast clearance and low background retention of [^18^F]FRPG throughout the body, this radiotracer holds promise for the imaging of system x_C_^-^ activity and treatment response monitoring in tumors of the thorax, abdomen, and head and neck. [^18^F]FRPG PET imaging provides a sensitive noninvasive measure of system x_C_^-^ and excellent properties for cancer imaging.

## Introduction

Cancer cells have unique metabolic demands that are required to support their rapid proliferation and progression [1]. Whilst conferring a survival advantage, this ‘hypermetabolic’ state introduces vulnerabilities that have been successfully exploited pharmacologically or through dietary restriction for anti-cancer therapy [2–5]. Despite the promise of these metabolic interventions in preclinical studies, their potential has yet to be fully realized in the clinic. In contrast, assessment of aberrant tumor glycolysis is routinely used for the clinical diagnosis and staging of cancer using [^18^F]2-fluoro-2-deoxy-D-glucose ([^18^F]FDG) positron emission tomography (PET) [6]. Despite its clinical utility, [^18^F]FDG uptake in healthy tissues with high glucose demand, such as the brain and at sites of inflammation, can complicate image interpretation.

The metabolic reprograming of tumors is not restricted to catabolic reactions that underpin glycolysis and central carbon metabolism; biosynthesis of DNA, lipids and proteins are additionally required to meet the needs of these rapidly dividing cells [7]. Amino acids are essential building blocks for macromolecular synthesis. Consequently, transformed cells frequently upregulate the expression of amino acid transporters on the plasma membrane, increase *de novo* amino acid synthesis, and modulate transamination pathways [8]. A range of radiolabeled natural and non-natural amino acid analogs have been developed to image the increased amino acid transporter expression found on cancer cells. In some instances, amino acid radiotracers have been reported to give improved tumor-to-background ratios over current standard of care imaging with [^18^F]FDG and may also provide prognostic information [9, 10]. Notable radiolabeled amino acids include: [^11^C]methionine, O-(2-^18^F-fluoroethyl)-L-tyrosine ([^18^F]FET) and 6-^18^F-fluoro-3,4-dihydoxy-L-phenylalanine ([^18^F]FDOPA), which have all seen a wide range of clinical applications [11–13].

**Figure 1:**
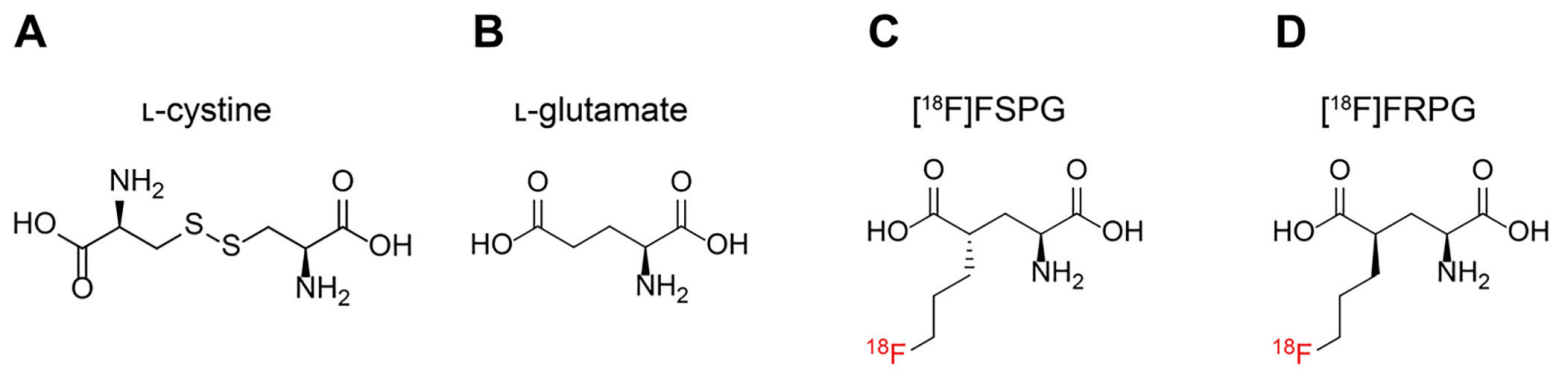
Molecular structures of system x_C_^-^ substrates L-cystine (A), L-glutamate (B), [^18^F]FSPG (C) and [^18^F]FRPG (D).

A consequence of the greater metabolic demand of cancer cells to sustain increased proliferation compared to non-transformed cells is the increased production of reactive oxygen species (ROS) in the mitochondria [14, 15]. To maintain cell viability, cancer cells upregulate endogenous antioxidants to counteract high ROS production. In addition to their anabolic role, amino acids can mediate redox homeostasis [16], with the amino acid transporter system x_C_^-^ being a key regulator of the tumor antioxidant response [17, 18]. System x_C_^-^ is a heterodimeric transporter consisting of a functional transporter (xCT/*SLC7A11*) and a membrane protein common to many amino acid transporters (CD98hc/*SLC3A2*) [19]. Functionally, the role of system x_C_^-^ is to exchange one molecule of extracellular L-cystine (Figure 1A) for one molecule of intracellular L-glutamate (Figure 1B). Following its uptake, cystine is rapidly reduced to cysteine, which is the rate limiting precursor for *de novo* glutathione (GSH) biosynthesis [20]. GSH is the most abundant thiol-containing antioxidant and necessary for ROS neutralization [21]. A continuous supply of GSH is therefore advantageous for cancer cells and is vital for the maintenance of redox homeostasis via cystine utilization [21]. The expression of system x_C_^-^ is regulated by key oncogenes and tumor suppressors, including KRAS [22], nuclear factor erythroid 2-related factor 2 (NRF2) [23] and p53 [24]. Increased system x_C_^-^ activity enhances cancer cell dependency on glucose, connecting the glycolytic and GSH metabolic pathways [25, 26]. Furthermore, the over-expression of this transporter renders cancer cells more resistant to chemotherapy [22, 24]. Consequently, several therapeutic strategies have been developed which target aberrant system x_C_^-^ activity [27, 28].

Given the importance of system x_C_^-^ in cancer cell survival, maintenance of redox homeostasis and its role in drug resistance, non-invasive imaging of this transporter’s activity may reveal vulnerabilities that can be exploited for anti-cancer therapy. (*S*)-4-(3-^18^F-fluoropropyl)-L-glutamate ([^18^F]FSPG; Figure 1C) was first examined as a system x_C_^-^ specific radiotracer and glutamate analog by Koglin *et al.* in preclinical tumor models [29]. Since then, [^18^F]FSPG has been used in pilot clinical trials for tumor detection in hepatocellular carcinoma, non-small cell lung cancer, intracranial malignancies and prostate cancer [30–33]. Additionally, we have previously shown that imaging with [^18^F]FSPG can monitor response to chemotherapy and predict drug resistance in models of ovarian cancer [34, 35]. System x_C_^-^ tolerates a broad range of non-natural amino acids based on the structures of either glutamate or cystine provided that a binding motif with an alpha amino-acid group and second carboxyl-group is maintained in suitable spatial distance. This promiscuity has been exploited by other fluorine-18 labelled PET radiotracers, including ^18^F-5-fluoro-aminosuberic acid ([^18^F]FASu) [36, 37] and [^18^F]hGTS13 [38].

[^18^F]FSPG contains two chiral centers, with four potential stereoisomers (2*R*/4*S*, 2*S*/4*S*, 2*R*/4*R* and 2*S*/4*R*) [39]. In biological systems chirality is an essential mediator of structure-activity relationships, given that ligand-binding domains have unique 3D conformations. As such, chirality guides enzyme-receptor interactions, drug efficacy and stability, compound toxicity, and transporter efficiency [40]. Here, we were motivated to investigate whether changes to the chirality of system x_C_^-^ substrates may improve tumor visualization when imaged with PET and report the initial preclinical evaluation of (*R*)-4-(3-^18^F-fluoropropyl)-L-glutamate ([^18^F]FRPG; Figure 1D), a stereoisomer of [^18^F]FSPG. Considering the established promiscuity of system x_C_^-^, we hypothesized that [^18^F]FRPG would be a specific substrate for this transporter, whilst minimizing reactivity with other intracellular enzymes. As well as probing [^18^F]FRPG target-specificity, we explored how the altered stereochemistry affected other important radiotracer properties, such as its metabolism and pharmacokinetics, with the goal to better understand system x_C_^-^ function in the context of cancer. To elucidate any advantages or limitations exhibited by [^18^F]FRPG as a system x_C_^-^ radiotracer, comparison experiments to the established radiotracer [^18^F]FSPG were conducted throughout in mouse models of cancer and acute inflammation of the lung.

## Methods

### Cell culture

Human cancer H460 and A549 cells were obtained from AddexBio Technologies, H460 Fluc cells from PerkinElmer, PC-3 and VCap cells from ATCC, and A2780 cells from Sigma Aldrich. H460, H460 Fluc and A2780 cells were cultured in RPMI 1460 (Sigma-Aldrich); PC-3 were cultured in F-12 K Nutrient Mixture with glutamine (ThermoFisher Scientific); VCap cells were cultured in Dulbecco’s MEM with GlutaMAX (ThermoFisher Scientific); and A549 cells were cultured in DMEM (Sigma-Aldrich). All media were supplemented with 10% fetal bovine serum (ThermoFisher Scientific). H460 Fluc, A549 and A2780 cells were additionally supplemented with 100 U.mL^−1^ penicillin and 100 μg.mL^−1^ streptomycin (Sigma-Aldrich). All cells were maintained at 37 °C and 5% CO_2_. Cell lines were authenticated by STR profiling.

### Radiotracer cell uptake and efflux

H460 cells were seeded into 48-well plates 48 h prior to uptake and efflux studies at a density of 3 × 10^4^cells/well. Cell density was determined using a Neubauer cell chamber and normalized to 100,000 cells/well at the time of the assay. For all cell uptake and efflux experiments, fresh PBS with 0.1% BSA containing ~0.25 MBq of [^18^F]FRPG or [^18^F]FSPG were added to wells (total volume 250 μL/well) over a predetermined time course. Cells were maintained at 37 °C and 5% CO_2_ throughout the uptake or efflux experiment. For competition studies, inhibitors (p-carboxyphenylglycine (CPG) and L-trans-pyrrolidine-2,4-dicarboxylic acid), transporter substrates (FSPG, L-glutamate, and D-glutamate) and non-transporter substrates (L-aspartate and D-aspartate) were co-incubated with [^18^F]FRPG at a final concentration of one mM. For efflux experiments, following 30 min uptake, exogenous radioactivity was removed, with cells washed twice in 0.5 mL PBS, and fresh PBS 0.1% BSA added for 30 minutes. Cells were lysed by the addition of 0.5 mL 1 N NaOH. The amount of radioactivity in the samples was determined in a gamma counter (Wizard 3, Perkin Elmer) and expressed as a percentage of the administered dose per 100,000 cells.

### Cell treatments

A2780 cells were seeded at a density of 5 × 10^5^ cells per well on a 6-well plate, 24 h prior to uptake studies. Fresh medium was provided 1-2 hours prior to drug treatments. Tert-butyl hydroperoxide (TBHP) was administered 1 hour prior to assay at a final concentration of 200 μM. N-acetylcysteine (NAC) was added 1 hour prior to TBHP administration (2 hours total treatment duration) at a final concentration of 5 mM. Following a 60 min uptake period, cells were processed as described previously [34].

### Western blot

Western blot analysis was carried out using an iBind Flex system (ThermoFisher Scientific) for primary and secondary antibody immunoblotting. For cell lysate collection, H460 cells were seeded in 6-well plates, 24 h prior to harvesting, at a density of 1.5 × 10^6^/mL in 2 mL media. Cells were placed on ice, washed three times with ice-cold PBS and lysed in 100 μL RIPA buffer containing 1× protease and phosphatase inhibitors (ThermoFisher Scientific). Collected lysates were then centrifuged at 15,000 × *g* at 4 °C for 10 min. Cell debris was removed and the supernatant aliquoted to avoid freeze-thaw cycles of lysates. For *ex vivo* sample preparation, tissues were dissected immediately following sacrifice, snap frozen in liquid nitrogen, and stored at -80 °C. Frozen tissues were next added to pre-chilled Matrix tubes containing 1.4 mm ceramic beads and RIPA buffer containing 1 × protease and phosphatase inhibitors. Samples were lysed by rapid shaking using a high-speed benchtop reciprocating homogeniser cooled to 4 °C (Precellys 24 homogeniser, Bertin Instruments). The lysates were centrifuged at 15,000 × *g* at 4 °C for 10 min and the supernatant collected for analysis. The protein content in each lysate was determined using a BCA assay kit and 20 μg of protein was loaded into each well.

For sample preparation, NuPAGE LDS Sample Buffer and NuPAGE Reducing Agent were added to 20 μg of cell lysates in a total volume of 20 μL. Samples were then vortexed and heated to 70 °C for 10 min to denature the proteins. When loading the samples, one well per gel was always reserved for a protein ladder (Magic Marker, ThermoFisher Scientific; SeeBlue™ Plus2, ThermoFisher Scientific). Following loading, 10% polyacrylamide gels were run at 140 V until the dye front ran to the bottom of the gel. Proteins were then transferred from the gel to a polyvinylidene fluoride (PVDF) membrane using a Trans-Blot Turbo Transfer System (Bio-Rad Laboratories) according to the manufacturer’s instructions. After transfer, an iBind Flex Western system (Invitrogen) was used for antibody binding and detection following the manufacture’s guidelines. Blots were probed for xCT (1:1000 dilution; Cell Signaling Technology). Actin was used as a loading control for all experiments, with a horseradish peroxidase (HRP) linked anti-rabbit IgG secondary antibody (1:200 dilution; Cell Signaling Technology). After antibody incubations, membranes were removed from the iBind Flex system and washed in 50 mL of trisbuffered saline with 0.1% Tween 20 (TBST), for 5 min, 5 times on a shaker (Stuart gyratory rocker SSL3). To visualise proteins, 4 mL of Amersham™ ECL Prime Western Blotting Detection Reagent (GE Healthcare) was added to each membrane, in the dark, for 1 min. Images were taken using an iBright CCD camera (Invitrogen). Images were always acquired within the linear range of the camera to prevent the overexposure of any blots.

### Animal studies

All animal experiments performed in the U.K. were in accordance with the United Kingdom Home Office Animal (scientific procedures) Act 1986. Animal experiments performed in Germany were conducted in compliance with the current local laws concerning animal protection and welfare and received local approval. Experiments were performed unblinded.

### Subcutaneous tumor models

For biodistribution studies, 5 × 10^6^ PC-3, VCap, H460 or A549 cancer cells in 100 μL PBS were injected subcutaneously into female NMRI nude mice aged 6-9 weeks (Taconic). For imaging, 5 × 10^6^ A549 and H460 cancer cells in 100 μL PBS were injected subcutaneously into female Balb/C nu/nu mice aged 6-9 weeks (Charles River Laboratories). Tumor growth was monitored using an electronic caliper and the volume calculated using the following equation: volume = ((π/6) × h × w × l), where h, w and l represent, height, width and length, respectively. Tumor size was monitored daily, with studies taking place when tumor volume reached ~100 mm^3^. Approximately 3 MBq of radiotracer was injected *via* the tail vein of anaesthetized mice (*n* = 3 per group). All mice were maintained under anesthesia and warmed to 37 °C for the duration of the experiment. For biodistribution studies, animals were sacrificed by cervical dislocation at specific time points over a period of 240 min, with tissues excised, weighed, and radioactivity subsequently determined on a gamma counter (Wizard 3, Perkin Elmer). The average of three standards equaling the injected dose were used to determine the percentage injected dose in each organ of interest. Radioactivity was normalized per gram of tissue (% ID/g). For quantification of radiotracer retention in tumors imaged by PET/CT, tumor volumes of interest were constructed from 2D regions drawn manually using the CT image as reference. Data were expressed as %ID/g, assuming a tissue density of one g per mL.

### Orthotopic lung tumor inoculation

1 × 10^6^ NCI-H460 FLuc cells were administered by a non-invasive intratracheal technique into the lungs of female NSG mice aged 6-9 weeks (Charles River Laboratories) based on previously described methods [41, 42]. Mice were anaesthetized with isoflurane (2-2.5% in O_2_) and transferred onto a vertical board where they were suspended in an upright position by their upper incisors. Using a Leica M125 stereomicroscope (Leica Microsystems), the tongue was moved to one side to expose the vocal cords and the entrance to the trachea, where a plastic 20 G i.v. catheter, attached to a 1 mL syringe was inserted. Approximately 50 μL of air was first injected into the lung to inflate it prior to the administration of 20 μL of cell suspension in PBS. Tumor growth was monitored through bioluminescent imaging. For further details see Supplemental Methods, Figures and Tables.

### Doxil treatment of mice bearing ovarian cancer xenografts

5 × 10^6^ A2780 cancer cells in 100 μL PBS were injected subcutaneously into female Balb/C nu/nu mice aged 6-9 weeks (Charles River Laboratories). Tumor growth was monitored as described above, with imaging taking place once the tumors reached ~100 mm^3^. Immediately following [^18^F]FRPG PET/CT imaging, mice were randomized and received a single intraperitoneal injection of 10 mg/kg Doxil (Caelyx, Janssen). [^18^F]FRPG PET/CT imaging was repeated 24 h after the treatment with Doxil.

### Lipopolysaccharide-induced lung inflammation

Inflammation was induced in female Balb/C mice (aged 6-9 weeks; Charles River Laboratories) through intratracheal inhalation of 50 μL lipopolysaccharide (LPS; 1.5 mg/kg; from Escherichia coli O55:B5; Sigma-Aldrich), administered using the same procedure as the orthotopic tumor cell inoculation. Mice in the control group were treated with 50 μL PBS. Imaging was performed 24 h after treatment.

### Radiotracer production and identification

[^18^F]FRPG and [^18^F]FSPG were synthesized from their corresponding naphthalenesulfonyl precursors (Life Molecular Imaging) using a GE FASTlab automated synthesis module using a previously reported method [43]. The analysis and identification of [^18^F]FRPG and [^18^F]FSPG was also conducted as reported previously [43]. For a detailed description of precursor and ^19^F reference standard syntheses, along with their corresponding spectra, see Supplementary Information: Synthesis and Spectra. The radiochemical purity and molar activity of [^18^F]FRPG and [^18^F]FSPG were analysed by pre-column derivatization with ortho-phthalaldehyde reagent (OPA). 20 μL [^18^F]FRPG or [^18^F]FSPG solution was added to 20 μL OPA reagent followed by addition of 80 μL PBS. The mixture was incubated for 5 min at room temperature and then analysed by HPLC. Next, the appropriate ^19^F standard was added to the OPA/tracer reaction mixture and left for 15 min before further analysis by HPLC. Co-elution of the ^19^F standard OPA-adduct UV peak with the corresponding ^18^F OPA-adduct radioactive peak confirmed the identity of both [^18^F]FRPG and [^18^F]FSPG (Figure S1). HPLC chromatograms showing the different retention times for the [^18^F]FRPG-OPA and [^18^F]FSPG-OPA adducts with co-elution of their corresponding ^19^F reference compounds can be found in the supplemental information (Figure S2).

### PET/CT imaging

For tumor imaging studies, mice were maintained under isoflurane anesthesia (1.5-2% in O_2_) at 37 °C during tail vein cannulation and imaging. For subcutaneous tumor imaging, dynamic PET scans were acquired on a Mediso NanoScan PET/CT system (1-5 coincidence mode; 3D reconstruction; CT attenuation-corrected; scatter corrected) using a four-bed mouse hotel (Mediso) [44]. Images were acquired for 60 min following a bolus intravenous injection of ~3 MBq [^18^F]FRPG or [^18^F]FSPG (100 μL) through a tail vein cannula. For serial imaging studies using both radiotracers, mice were randomized to first receive either [^18^F]FSPG or [^18^F]FRPG. CT images were acquired for anatomical visualization (480 projections; helical acquisition; 55 kVp; 600 ms exposure time). For orthotopic tumor imaging studies, mice were intravenously injected with ~3 MBq of radiotracer, with PET scans acquired for 20 min following a 40 min uptake period. Animals were maintained under anesthesia from the time of radiotracer injection until the completion of imaging. CT images were acquired for anatomical visualization (720 projections; semicircular acquisition; 55 kVp; 600 ms exposure time) using a conventional single mouse imaging bed. All images were reconstructed using Tera-Tomo 3D (Mediso) with 4 iterations, 6 subsets, and 0.4 mm isotropic voxel size. Radiotracer concentration was quantified using VivoQuant software (v 2.5, Invicro Ltd). Tumor volumes of interest were constructed from 2D regions drawn manually using the CT image as reference. Data were expressed as percent injected dose per gram of tissue (% ID/g).

### Histology

For *ex vivo* H&E staining and immunohistochemistry of lung tissue, please see Supplemental Methods, Figures and Tables.

### *In vivo* metabolite analysis

*In vivo* metabolism of [^18^F]FRPG and [^18^F]FSPG in blood, liver, pancreas, urine, and A549 tumors samples were analyzed by radio-HPLC 60 min after injection of ~10 MBq of radiotracer. For further details, see Supplemental Methods, Figures and Tables.

### Statistics

Statistical analysis was only performed using GraphPad Prism (v.8.0) on data sets acquired from three or more biological replicates, acquired on separate days. All data were expressed as the mean ± one standard deviation (SD). Statistical significance was determined using either unpaired or paired two-tailed Student’s t-test. For analysis across multiple samples, 1-way analysis of variance (ANOVA) followed by t-tests multiple comparison correction (Tukey method) were performed. Data was considered as significant if *P* < 0.05.

## Results

### The chiral integrity of [^18^F]FRPG and [^18^F]FSPG are maintained during their preparation

[^18^F]FSPG and [^18^F]FRPG were obtained in PBS in 37.8 ± 4.0% (*n* = 4) and 38.4 ± 2.6% (*n* = 3) radiochemical yields, respectively. For quality-control, the radiotracers were reacted with OPA, which enabled their UV detection by HPLC ([^18^F]FSPG-OPA and [^18^F]FRPG-OPA). Addition of non-radioactive reference compounds to the OPA/radiotracer reaction mixture and their co-elution with the radioactive peak confirmed their identity and gave a radiochemical purity of >96% (Figure S1). As both [^18^F]FSPG-OPA and [^18^F]FRPG-OPA gave similar retention times, HPLC conditions were further optimized to separate the two radiotracer-OPA adducts, eluting at 14.5 min and 13.2 min for [^18^F]FRPG-OPA and [^18^F]FSPG-OPA, respectively. Separation of the two radiotracer’s OPA adducts and co-elution of cold standards under these conditions further confirmed the separate identities of the two radiotracers and the absence of epimerization during the radiotracer syntheses (Figure S2).

### [^18^F]FRPG is a substrate for system x_C_^-^

**Figure 2:**
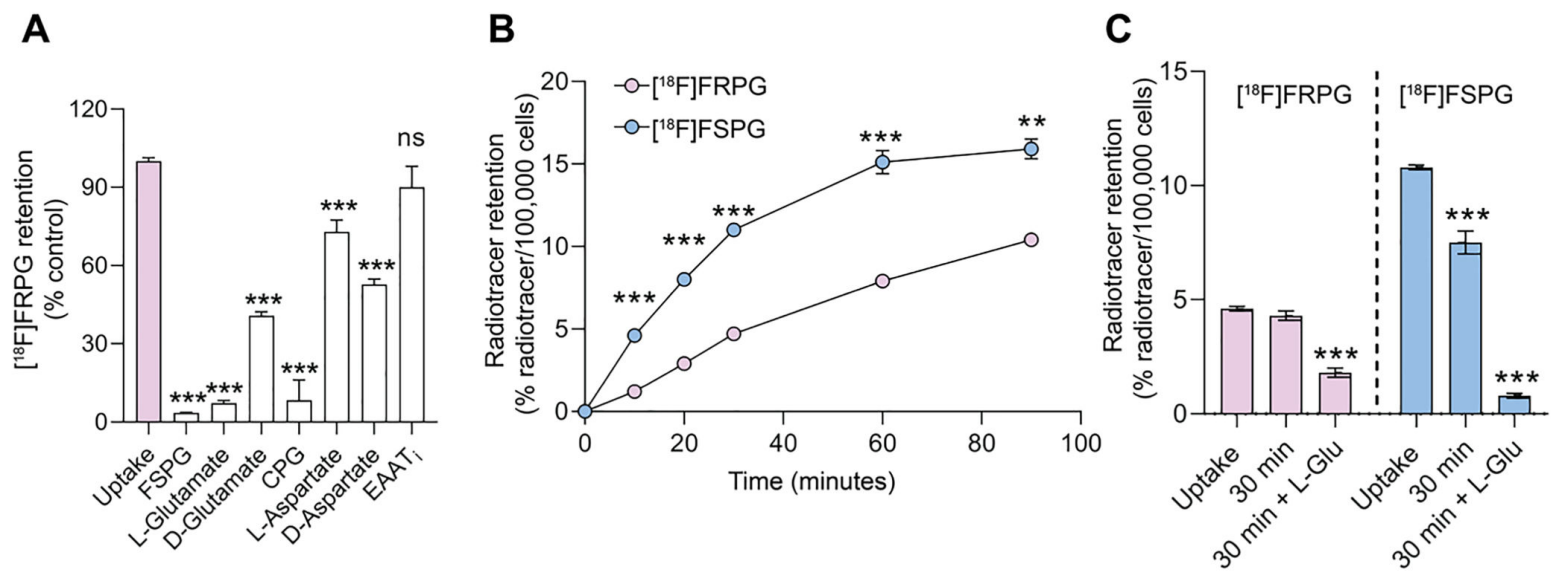
Cellular characterization of transporter specificity, uptake, and efflux of [^18^F]FRPG. **(A)** Competition studies of [^18^F]FRPG in H460 lung cancer cells. All inhibitors and substrates were added at the same time as [^18^F]FRPG and incubated for 30 min. Differences in [^18^F]FRPG cell uptake were compared to vehicle-treated control cells. CPG, p-carboxyphenylglycine; EAATi, L-trans-pyrrolidine-2,4-dicarboxylic acid. (**B**) Time course [^18^F]FRPG and [^18^F]FSPG uptake in H460 cells over 90 minutes. **(C)** [^18^F]FRPG and [^18^F]FSPG efflux with or without the addition of 1 mM L-glutamate following preloading of cells with radiotracer for 30 min. ns, not significant; **, *P* < 0.01; ***, *P* < 0.001; *n* = 3. Data are presented as mean ± SD.

To determine the specificity of [^18^F]FRPG in H460 cancer cells, we co-incubated [^18^F]FRPG for 30 min with non-radioactive substrates of system x_C_^-^ present in high molar excess (1 mM; Figure 2A). Baseline [^18^F]FRPG uptake in H460 cells was reduced by 94% in the presence of L-glutamate, from 4.7 ± 0.1% radioactivity/100,000 cells in untreated cells to 0.3 ± 0.04% radioactivity/100,000 cells (*P* < 0.0001; *n* = 3). A 59% reduction in [^18^F]FRPG was measured in the presence of the non-natural D-glutamate stereoisomer (1.9 ± 0.07% radioactivity/100,000 cells; *P* < 0.0001; *n* = 3), with the highest level of blocking observed with [^19^F]FSPG (96%; 0.2 ± 0.01% radioactivity/100,000 cells; *P* < 0.0001; *n* = 3). Furthermore, the xCT inhibitor CPG reduced [^18^F]FRPG uptake by 92% when compared to untreated cells (0.39 ± 0.37% radioactivity/100,000 cells; *n* = 3; *P* < 0.0001). Interestingly, non-system x_C_^-^substrates L-aspartate and D-aspartate reduced [^18^F]FRPG uptake by 27% and 47%, respectively, to 3.4 ± 0.2% radioactivity/100,000 cells and 2.5 ± 0.1% radioactivity/100,000 cells (*P* = 0.0006 for L-aspartate, *P* < 0.0001 for D-aspartate; *n* = 3). However, when [^18^F]FRPG was incubated with an inhibitor of the excitatory amino acid transporter (EAAT), there was no significant change in radiotracer retention (5.6 ± 0.4% ID/mg protein in control cells compared to 5.1 ± 0.7% ID/mg protein in treated cells; *P* = 0.3; *n* = 3).

To further confirm specificity of [^18^F]FRPG for system x_C_^-^, light chain xCT protein expression was reduced in H460 cells by siRNA, as confirmed by western blot (Figure S3A). 48h of siRNA treatment decreased xCT protein expression by 52% compared to untreated cells once normalised for actin, whereas no change was seen with scrambled control siRNA. [^18^F]FRPG was reduced by 52% in xCT siRNA cells compared to cells transfected with control siRNA (4.04% ID/mg protein *versus* 8.34% ID/mg protein; *n* = 3 technical repeats; Figure S3B). Similarly, we observed a 40% reduction in [^18^F]FSPG retention in cells transfected with xCT siRNA, compared to control siRNA (6.5% ID/mg protein and 10.8% ID/mg protein, respectively; *n* = 3 technical repeats).

### Amino acid stereochemistry affects radiotracer uptake and efflux kinetics

In cell culture, addition of [^18^F]FSPG to H460 cells resulted in rapid cellular uptake over the initial 60 min, reaching 15.1 ± 0.7% radioactivity/100,000 cells (*n* = 3). No further increase in cell retention was observed for [^18^F]FSPG between 60-90 min. Conversely, [^18^F]FRPG was taken into cells at a slower linear rate over the 90 min time course. Consequently, cell-associated radioactivity was significantly lower at 90 min with [^18^F]FRPG compared to [^18^F]FSPG (10.4 ± 0.1% radioactivity/100,000 cells and 15.9 ± 0.6% radioactivity /100,000 cells, respectively; *P* = 0.007; *n* = 3; Figure 2B). Intracellular retention of radiolabeled glutamate analogs is the product of their uptake *via* system x_C_^-^ and any subsequent efflux. We next probed radiotracer efflux from cells after a 30 min uptake period. A further 30 min after removal of exogenous radioactivity, intracellular [^18^F]FSPG was reduced by 30.6% (*P* < 0.0001; *n* = 3), whereas there was no significant decrease in cellular retention for [^18^F]FRPG (*P* = 0.17; *n* = 3). Addition of 1 mM glutamate in the radiotracer-free media resulted in extensive efflux of cell-associated radioactivity, with intracellular radioactivity reduced by 93% (*P* < 0.0001; *n* = 3) and 61% (*P* < 0.0001; *n* = 3) for [^18^F]FSPG and [^18^F]FRPG, respectively (Figure 2C).

### [^18^F]FRPG is rapidly cleared from non-target tissues

**Figure 3:**
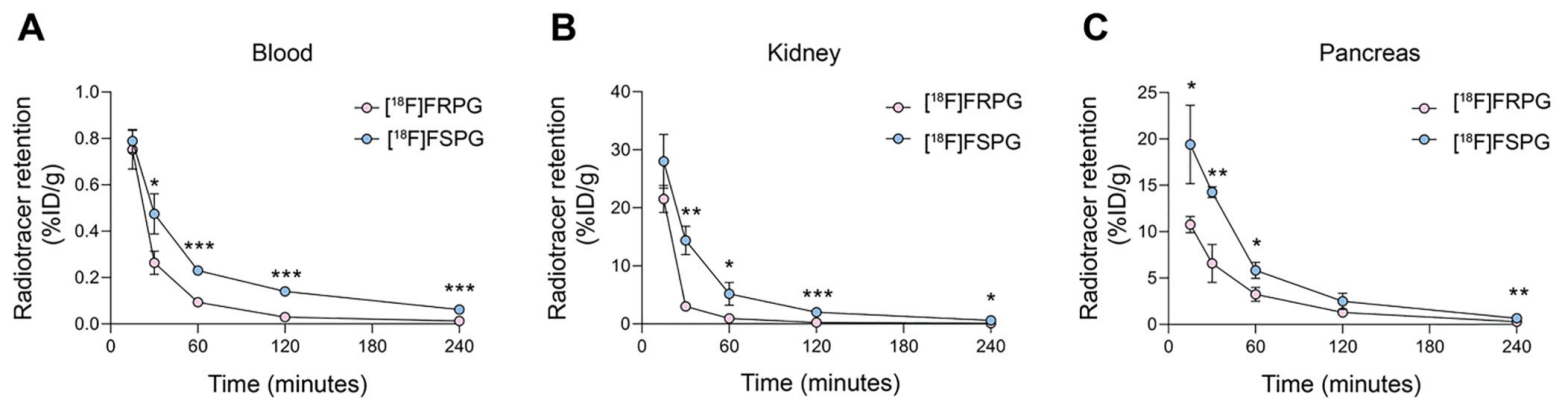
[^18^F]FRPG and [^18^F]FSPG pharmacokinetics. Time course biodistribution of [^18^F]FRPG and [^18^F]FSPG in mice over 240 min in **(A)** blood, **(B)** kidney and **(C)** pancreas. *, *P* < 0.05; **, *P* < 0.01; ***, *P* < 0.001. Data are presented as mean ± SD for three separate mice.

Given that the stereochemistry of fluorinated glutamate analogs affected system x_C_^-^ tumor cell uptake and retention kinetics, we next investigated whether [^18^F]FRPG *in vivo* pharmacokinetics differed from [^18^F]FSPG in mice bearing H460 subcutaneous tumors (Figure 3A-C). Both radiotracers were characterized by rapid extraction from the blood and elimination *via* the urinary system. Whilst initial tissue delivery of [^18^F]FRPG was similar to [^18^F]FSPG, [^18^F]FRPG was subsequently cleared from non-target tissues at a faster rate than [^18^F]FSPG. At 15 min post injection (p.i), there was no difference in radioactivity concentration between [^18^F]FRPG and [^18^F]FSPG in the blood (0.75 ± 0.08% ID/g *vs*. 0.79 ± 0.04% ID/g, respectively; *P* = 0.54; *n* = 3) or kidney (28.0 ± 4.64% ID/g *vs*. 21.5 ± 2.34% ID/g, respectively; *P* = 0.03; *n* = 3). By 30 min p.i, however, the concentration of [^18^F]FRPG in the blood was half that of [^18^F]FSPG (0.26 ± 0.05% ID/g *vs*. 0.47 ± 0.09% ID/g, respectively; *P* = 0.02; *n* = 3), with [^18^F]FRPG retention almost 5-fold lower than [^18^F]FSPG in the kidney (3.02 ± 0.34% ID/g *vs*. 14.4 ± 2.45% ID/g, respectively; *P* = 0.02; *n* = 3). The amount of [^18^F]FRPG in the pancreas, an organ with high xCT expression [45, 46], remained lower than [^18^F]FSPG over the first 60 min (3.2 ± 0.8% ID/g *vs*. 5.8 ± 0.9% ID/g respectively at 60 min; *P* = 0.02; *n* = 3). Faster washout of [^18^F]FSPG from the pancreas resulted in similar levels of retention as [^18^F]FRPG at 120 min (2.5 ± 0.88% ID/g *vs*. 1.28 ± 0.19% ID/g; *P* = 0.08; *n* = 3).

### [^18^F]FRPG PET generates high-contrast tumor images

**Figure 4:**
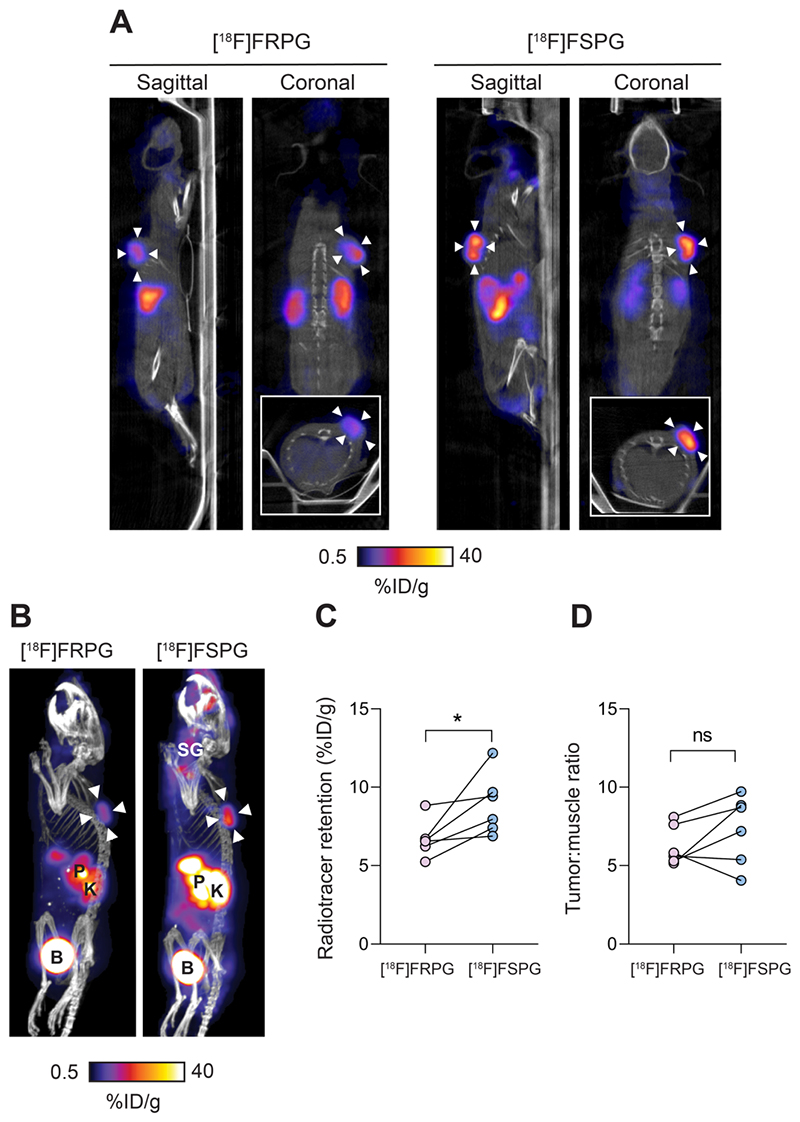
Evaluation of [^18^F]FRPG and [^18^F]FSPG in tumor-bearing mice. **(A)** Representative single slice sagittal, coronal and axial (inset) PET/CT images in A549 subcutaneous tumor-bearing mouse 40-60 min after injection of ~3 MBq [^18^F]FRPG or [^18^F]FSPG. Imaging with each radiotracer was performed 24 h apart in the same mouse. White arrowheads indicate the tumor margins. **(B)** Representative PET/CT maximum intensity projections from the same mouse. White arrowheads indicate the tumor margins. SG, salivary glands; P, pancreas; K, kidney; B, bladder. **(C)** Image-derived quantification of radiotracer retention in matched A549 tumors imaged 24-48 h apart. Data points represent individual animals. *, *P* < 0.05. **(D)** Image-derived tumor-to-muscle ratios for [^18^F]FRPG and [^18^F]FSPG. Data points represent individual animals. ns, not significant.

To better understand differential tumor retention of system x_C_^-^ radiotracers, [^18^F]FRPG and [^18^F]FSPG PET imaging was compared in the same A549 tumor-bearing mice, imaged 24-48 h apart. Mice were randomly assigned to be imaged with [^18^F]FRPG or [^18^F]FSPG first. Representative 40-60 min single-slice [^18^F]FRPG and [^18^F]FSPG images and maximum-intensity projections (MIPs) are shown in Figure 4A and 4B for comparison. [^18^F]FRPG radiotracer distribution matched *ex vivo* measurements, as characterized by rapid clearance from most healthy tissues by 60 min. Image-derived [^18^F]FRPG radioactivity was 25% lower in A549 tumors than [^18^F]FSPG, at 6.7 ± 1.18% ID/g and 8.9 ± 1.95% ID/g, respectively (*P* = 0.034; *n* = 6; Figure 4C). Due to faster [^18^F]FRPG washout kinetics in healthy tissue, however, there was no difference in the tumor-to-muscle ratios for both tracers (6.26 ± 1.26 *vs*. 7.32 ± 2.21 for [^18^F]FRPG and [^18^F]FSPG, respectively; *P* = 0.22; *n* = 6; Figure 4D).

**Figure 5:**
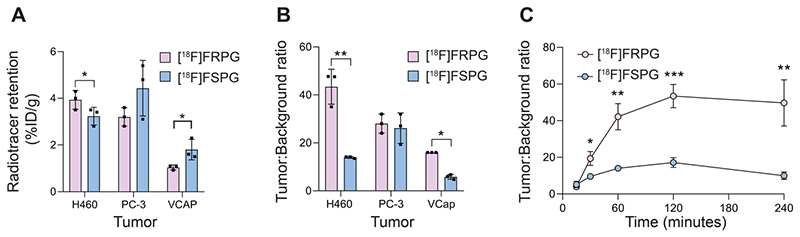
[^18^F]FRPG and [^18^F]FSPG tumor retention and contrast. **(A)** Radiotracer retention in subcutaneously grown H460, PC-3 and VCAP tumors at 60 min post injection, determined by *ex vivo* biodistribution. **(B)** Corresponding tumor-to-blood ratios 60 min post injection. **(C)** Time course [^18^F]FRPG and [^18^F]FSPG H460 tumor-to-blood ratios over 240 min. Data are presented as mean ± SD for three separate mice. *, *P* < 0.05; **, *P* < 0.01; ***, *P* < 0.001.

*Ex vivo* biodistribution studies were used to further assess tumor radiotracer retention in prostate and lung cancer xenografts. At 60 min p.i. [^18^F]FRPG retention was lower than [^18^F]FSPG in prostate VCAP tumors, at 1.0 ± 0.11% ID/g *versus* 1.8 ± 0.44% ID/g, respectively (*n* = 3; *P* = 0.04; Figure 5A). Radiotracer retention, however, were similar in PC-3 prostate tumors (3.2 ± 0.4% ID/g for [^18^F]FRPG *vs*. 4.4 ± 1.19% ID/g for [^18^F]FSPG; *P* = 0.16; *n* = 3) and H460 small cell lung cancer tumors (3.9 ± 0.4% ID/g for [^18^F]FRPG *vs*. 3.2 ± 0.38% ID/g for [^18^F]FSPG; *P* = 0.09; *n* = 3; Figure 5A). Given the rapid washout of [^18^F]FRPG from non-target tissues, excellent tumor-to-blood ratios ≥10 were achieved, which were either higher or equal to those of [^18^F]FSPG (Figure 5B). [^18^F]FRPG tumor-to-blood ratios were approximately 3-fold higher than [^18^F]FSPG in VCAP (16.0 ± 0.0 *vs*. 5.9 ± 1.02, respectively; *P* = 0.0034; *n* = 3) and H460 tumors (43.4 ± 7.3 *vs*. 14.0 ± 0.41, respectively; *P* = 0.02; *n* = 3). There was no significant difference in the radiotracer tumor-to-blood ratios for PC-3 tumors (28.0 ± 4.0 *vs*. 26.1 ± 6.3 for [^18^F]FRPG and [^18^F]FSPG, respectively; *P* = 0.65; *n* = 3;). Time course radiotracer retention in H460 tumors was further examined over 4 h. Here, tumor-to-blood ratios peaked at 120 min for both radiotracers, which was 53.4 ± 6.3 for [^18^F]FRPG and 17.2 ± 2.7 for [^18^F]FSPG, a three-fold difference (*n* = 3; *P* = 0.0008; Figure 5C).

To confirm the specificity of [^18^F]FRPG for system x_C_^-^ in living subjects, mice bearing subcutaneous H460 tumors were imaged with [^18^F]FRPG PET/CT at baseline and then reimaged 24 h later following the intratumoral administration of the xCT inhibitor IKE. Representative sagittal, coronal and axial PET/CT images, 40-60 min post injection of [^18^F]FRPG, are shown in Supplementary Fig. S4. With IKE treatment, [^18^F]FRPG tumor retention was reduced 42%, falling from 3.6 ± 1.3% ID/g in the tumors of untreated mice to 1.8 ± 0.5% ID/g (*P* = 0.05, paired one-way t test; *n* = 6). In one animal, tumor retention was reduced 87%, which was thought to be a result of excellent drug delivery to the tumor.

### Stereochemistry differentially affects metabolic stability of fluorinated glutamate analogs

**Figure 6:**
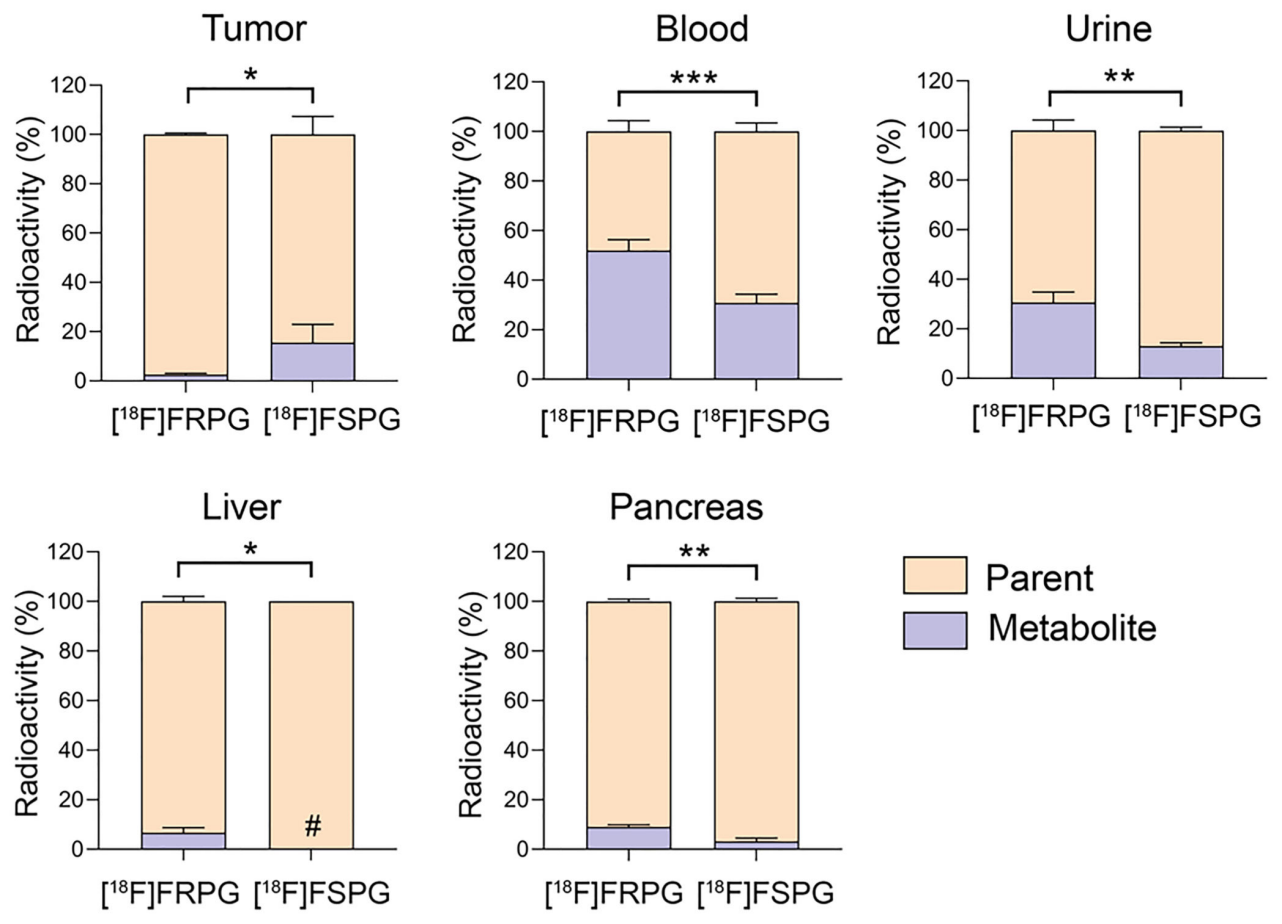
[^18^F]FRPG and [^18^F]FSPG *in vivo* metabolism. Percentage of parent radiotracer and radio-metabolite from the tumor, blood, liver, pancreas and urine for [^18^F]FRPG and [^18^F]FSPG. *, *P* < 0.05; **, *P* < 0.01; ***, *P* < 0.001. Data are presented as mean ± SD (*n* = 3-5). #, metabolite below detection threshold.

Tissue metabolite analysis was performed in A549 tumor-bearing mice at 60 min p.i. and analyzed by radio-HPLC. Both [^18^F]FRPG and [^18^F]FSPG were differentially metabolized *in vivo* to a single species (Figures S5 and S6). In A549 tumors, both [^18^F]FRPG and [^18^F]FSPG were predominantly present as the parent compound, with a higher percentage of the parent radiotracer present with [^18^F]FRPG (98%) compared to [^18^F]FSPG (85%; *P* = 0.011; *n* = 4-5; Figure 6). Conversely, lower levels of intact [^18^F]FRPG compared to [^18^F]FSPG was present in the urine (*P* = 0.002; *n* = 3), liver (*P* = 0.030; *n* = 3-4;), pancreas (*P* = 0.003; *n* = 3), and in blood (*P* = 0.0008; *n* = 3-4), where ~50% of [^18^F]FRPG was converted to the unknown metabolite by 60 min (Figure 6 and Table S1).

### [^18^F]FRPG retention is significantly higher in tumor tissue compared to inflammation

**Figure 7:**
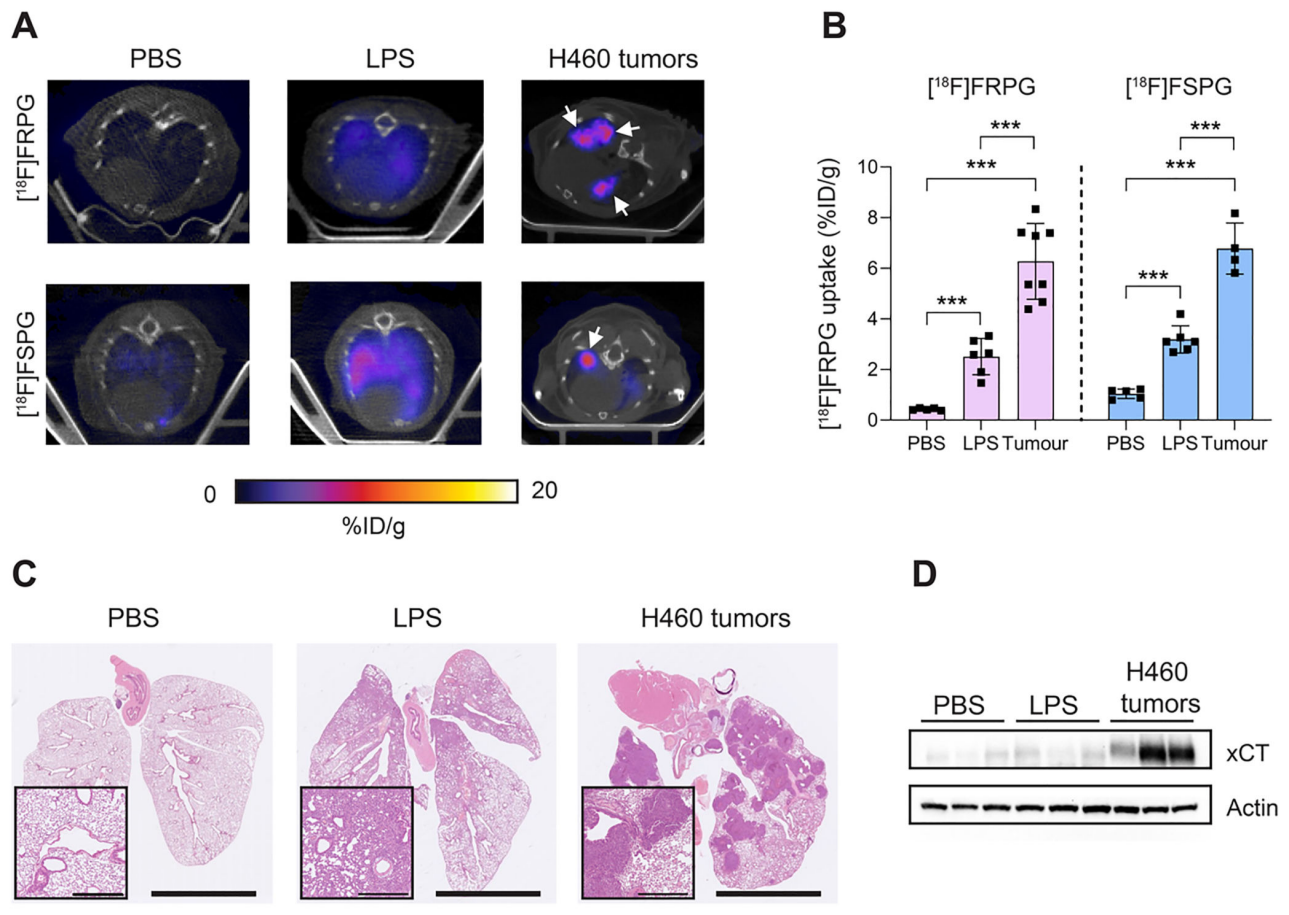
Non-invasive [^18^F]FRPG and [^18^F]FSPG PET/CT imaging in healthy, inflamed and tumor-containing lungs. **(A)** Single slice axial PET/CT images 40-60 min p.i. showing [^18^F]FRPG and [^18^F]FSPG retention in vehicle-treated mice, inflamed lungs as a result of 24 h LPS treatment, and retention in orthotopic H460 lung tumors. White arrows indicate the tumor. **(B)** Image-derived quantification of [^18^F]FRPG and [^18^F]FSPG retention in the lung. Data are presented as mean ± SD. For PBS and LPS groups, scatter plots represent individual animals. For the tumor group, scatter plots represent values from individual lesions from two animals. ***, *P* < 0.001. **(C)** Representative H&E staining of lung tissue following treatment with PBS, LPS or with orthotopic H460 tumors. Main images are shown at 0.46× magnification (scale bars, 5 mm) and inserts at 5× magnification (scale bars, 500 μm). **(D)** Expression of xCT in control, LPS treated, and H460 tumor tissue examined by western blot. Actin was used as a loading control.

Next, we compared [^18^F]FRPG and [^18^F]FSPG retention in the lungs of healthy mice to those with acute lung inflammation and mice bearing orthotopic lung tumors (Figure 7A). [^18^F]FRPG retention in the lungs was increased 5.8-fold following the induction of inflammation with LPS compared to PBS treatment alone (2.51 ± 0.71% ID/g and 0.43 ± 0.04% ID/g, respectively; *P* = 0.014; *n* = 5-6; Figure 7B). Tissue damage and immune cell influx with LPS treatment was confirmed by immunohistochemistry (Figure 7C and Figure S7, respectively). [^18^F]FRPG tumor retention in orthotopically grown H460 lung tumors (Figure 7A) was significantly higher than either the healthy or inflamed lung, with radioactivity reaching 6.78 ± 1.49% ID/g (14.6-fold and 2.5-fold higher, respectively; *P* < 0.0001; *n* = 8 lesions from 2 mice; Figure 7A and B). The MIPs further demonstrated that high-contrast lung tumor images can be obtained with [^18^F]FRPG (Figure S8). For comparison, [^18^F]FSPG was also evaluated in the same models of lung disease. There was a 3.1-fold increase in [^18^F]FSPG retention in the lungs of LPS treated mice (3.2 ± 0.54% ID/g) compared to control lungs (1.04 ± 0.19% ID/g; *P* < 0.0001; *n* = 5-6; Figure 7A and B). [^18^F]FSPG tumor retention increased 2.2-fold compared to LPS treated lungs and 6.5-fold compared to control tissue (6.8 ± 1.0% ID/g). There was no difference in [^18^F]FRPG and [^18^F]FSPG retention in the orthotopically grown H460 tumors (*P* = 0.53; *n* = 4-8). High [^18^F]FRPG and [^18^F]FSPG retention corresponded to elevated xCT expression in H460 orthotopic tumors compared to the lungs of both vehicle and LPS-treated mice (Figure 7D).

### [^18^F]FRPG retention is altered following the manipulation of the cellular redox environment

**Figure 8:**
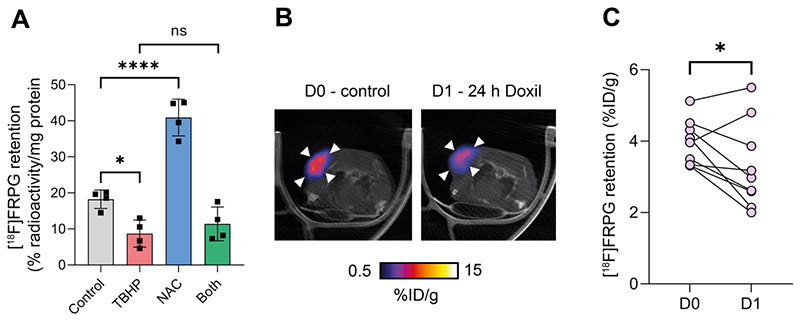
[^18^F]FRPG tumor retention is altered following manipulation of the redox environment. **(A)** intracellular retention of [^18^F]FRPG after treatment with the oxidant TBHP, antioxidant NAC, and a combined treatment of both. Data are presented as mean ± SD. Scatter plots represent independent experiments, performed in triplicate and presented as mean ± SD. TBHP, tert-butyl hydroperoxide; NAC, N-acetylcysteine. **(B)** Representative axial [^18^F]FRPG PET/CT images from matched mice receiving no Doxil (D0) and 24 hours of Doxil treatment (D1). White arrow heads represent the tumor outline. **(C)** Quantified [^18^F]FRPG tumor retention in matched animals before and 24 h after Doxil treatment. ns, not significant; *, *P* < 0.05; ****, *P* < 0.0001.

We have previously shown that [^18^F]FSPG retention in A2780 ovarian cancer cells was halved following oxidizing therapy (TBHP) and doubled with antioxidant treatment (NAC) [35]. To investigate whether drug-induced changes in the redox environment could be monitored with [^18^F]FRPG, we performed radiotracer uptake studies under the same conditions. Following the induction of oxidative stress by TBHP, [^18^F]FRPG retention was reduced from 18.2 ± 2.6% radioactivity/mg protein in control cells to 8.7 ± 3.8% radioactivity/mg protein, a 52% decrease (Figure 8A; *P* = 0.02; *n* = 4). Treatment with NAC resulted in a 2.2-fold increase in [^18^F]FRPG retention compared to control cells (40.9 ± 5.1% radioactivity/mg protein; *P* < 0.0001; *n* = 4). When NAC was given as a pretreatment to TBHP, [^18^F]FRPG retention remained at similar levels to TBHP treatment alone (11.4 ± 4.7% radioactivity/mg protein; *P* = 0.1; *n* = 4). *In vivo*, [^18^F]FRPG retention in A2780 xenografts was reduced by 15% 24h after treatment with Doxil (3.9 ± 0.6% ID/g and 3.3 ± 1.2% ID/g for control and 24 h Doxil treatment, respectively; *P* = 0.03, paired one-way *t* test; *n* = 9; Figure 7B and 7C). The observed decrease in [^18^F]FRPG tumor retention coincided with previous findings of Doxil-induced depletion of tumor GSH prior to a reduction in tumor volume [35].

## Discussion

The progression of neoplastic cells from pre-malignant lesions to primary invasive cancers and metastatic disease is accompanied by increasing levels of cellular oxidative stress [47]. Either through adaptive or evolutionary mechanisms, tumor cells respond to this insult by upregulating key antioxidant pathways to maintain redox homeostasis [48]. Upon treatment with oxidizing therapies (e.g. chemotherapy and radiotherapy), selective pressure often results in the regrowth of therapy-resistant disease characterized by further increases in tumor antioxidant capacity [49]. System x_C_^-^ can mediate this adaptive cellular response to oxidative stress [17, 28, 50]. A non-invasive readout of system x_C_^-^ activity therefore offers an important understanding into tumor behavior, with the potential to provide insight into drug response and resistance [51]. Here, we investigated whether the chirality of system x_C_^-^-specific radiotracers can be exploited for improved tumor imaging of system x_C_^-^ in cell models and living subjects.

Chirality plays an important role in living systems. Multiple chiral small molecules, including amino acids and sugars, exist; frequently, however, only one stereoisomer possesses biological activity. There has been extensive research into the effect of chirality on radiotracer pharmacokinetics, *in vivo* stability, and accumulation in non-target tissue [52–54]. Notably, ^18^F-labeled 4-fluoro-glutamine (4-FGln), has four stereoisomers which have been used to assess glutamine utilisation in various tumor types [52]. The synthesis and biological evaluation of the four ^18^F-labeled stereoisomers led to the identification of (2*S*,4*R*)-[^18^F]FGln as the lead-candidate radiotracer [55], which has subsequently been trialed in patients [56, 57]. Additionally, previous work examining the chirality of [^18^F]FASu, a system x_C_^-^ specific radiotracer, has shown the 2*S*-isomer to have superior imaging properties over the 2*R*-isomer [58].

Here, tumor cell retention of [^18^F]FRPG, a stereoisomer of the established system x_C_^-^ radiotracer [^18^F]FSPG, was highly selective for system x_C_^-^, demonstrated by >90% reduction in uptake in lung cancer cells upon inhibition of the antiporter. Interestingly, non-system x_C_^-^ substrates L-aspartate and D-aspartate also reduced [^18^F]FRPG retention by 27% and 47%, respectively (Figure 2A), unlike [^18^F]FSPG retention which displayed ~20% reduction when co-incubated with D-aspartate [29]. Excitatory amino acid transporters (EAATs) transport L-glutamate, L-aspartate and D-aspartate with similar affinity [29]. Whilst a reduction in cell-associated [^18^F]FRPG when incubated with high concentrations of aspartate may indicate transport mechanisms via non-system x_C_^-^ glutamate transporters such as EAATs, the reduction in cellular [^18^F]FRPG can similarly be explained by perturbations in system x_C_^-^ activity. A high molar excess of extracellular aspartate will likely block EAAT-mediated glutamate transport into the cell, thereby decreasing intracellular glutamate concentrations [59]. A reduction in the glutamate concentration gradient across the plasma membrane will consequently decrease system x_C_^-^ activity [60] and therefore [^18^F]FRPG cell retention, as we have shown previously with [^18^F]FSPG [35]. Additionally, no reduction in [^18^F]FRPG retention was observed when cells were treated with an EAAT inhibitor (Figure 2A), with [^18^F]FRPG efflux only evident following incubation with a system x_C_^-^ exchange partner upon removal of exogenous activity (Figure 2C). Indeed, [^18^F]FRPG retention was reduced following incubation of radiotracer-loaded cells with excess L-glutamate, providing further evidence of system x_C_^-^-mediated [^18^F]FRPG transport. Genetic modulation of xCT protein expression resulted in a corresponding decrease in both [^18^F]FRPG and [^18^F]FSPG cell retention, with pharmacological inhibition of system x_C_^-^ significantly reducing [^18^F]FRPG tumor-associated radioactivity *in vivo*. Taken together, [^18^F]FRPG appears to be exclusively mediated by system x_C_^-^.

The synthesis of all four stereoisomers of FSPG have recently been reported as clinical reference compounds, although their biological activity has not been assessed [39]. Here, we sought to understand the effect of system x_C_^-^ substrate chirality on transporter activity in cancer cells, using [^18^F]FRPG. In H460 cells grown in culture, the rate of both [^18^F]FRPG’s uptake and efflux was lower than [^18^F]FSPG, reflecting possible differences in the radiotracers’ affinity for system x_C_^-^. The stereoselectivity of system x_C_^-^ was further confirmed by differential [^18^F]FRPG cell retention in the presence of D-glutamate compared to L-glutamate (Figure 2A). *In vivo*, [^18^F]FRPG was rapidly and extensively taken up into tumors. Whilst tumor-associated [^18^F]FSPG radioactivity was the same or higher than [^18^F]FRPG, the lower-non-target tissue retention and faster blood clearance of [^18^F]FRPG (Figure 3) resulted in excellent tumor visualization, with tumor-to-background ratios of [^18^F]FRPG equal to or better than [^18^F]FSPG in the four tumor models evaluated (Figure 4 and 5). The low background retention in the abdomen and head and neck region should be beneficial for imaging small cancer lesions at these sites.

Similarly to tumors, [^18^F]FRPG retention was reduced compared to [^18^F]FSPG in organs that express high levels of system x_C_^-^ such as the pancreas and salivary glands [45, 46]. System x_C_^-^ is also expressed on cells of the immune system and upregulated following T-cell, B-cell and monocyte activation [61–63], and are [^18^F]FSPG-avid [38, 64]. Radiotracer retention in inflammatory cells may therefore confound interpretation of [^18^F]FRPG tumor uptake as a variety of activated immune cells reside in the complex tumor microenvironment. [^18^F]FRPG retention in orthotopic lung tumors, however, was 2.5-fold higher than inflamed lung tissue and 14.6-fold higher than normal lung tissue, indicating that tumor cell-specific signal will likely predominate.

Given the likely lower affinity of [^18^F]FRPG to system x_C_^-^, it was surprising that the tumor retention of [^18^F]FRPG and [^18^F]FSPG was similar in both H460 and PC-3 xenografts (Figure 5A). These findings suggest that radiotracer retention is governed by factors other than the affinity for system x_C_^-^ or the activity of this antiporter. Compound stereochemistry is a crucial factor in determining metabolic stability [65]. Previously, [^18^F]FSPG was reported to be metabolically stable *in vivo* [29, 66]. Our *in vivo* metabolism studies, however, revealed that both [^18^F]FRPG and [^18^F]FSPG were converted to a single radiolabeled metabolite which was more lipophilic than the parent compound (Figure S5 and S6). This is important as the imaging signal is the product of the uptake and efflux not only of the two parent radiotracers but also their corresponding metabolites. [^18^F]FSPG metabolism may have been overlooked in previous studies due to limitations in the analytical methods used (radio-thin layer chromatography) or differences in the biological system evaluated. Whilst metabolite concentrations remained low in A549 tumors (Figure 6), differences in the two stereoisomers’ metabolism may result in altered radiotracer retention.

Changes in metabolic profile and affinity for system x_C_^-^ may also explain differential radiotracer retention observed in healthy organs such as the pancreas, skin and salivary glands observed on imaging. The higher quantities of the [^18^F]FRPG metabolite in the blood and urine samples compared to its *S*-isomer suggest either elevated blood metabolism or quicker tissue clearance of this species. The latter may account for the lower levels of [^18^F]FRPG metabolite observed in tumor tissue, however radioactivity was not lost from the cell when system x_C_^-^ exchange substrates were absent (Figure 2C), appearing to rule out this possibility. Establishing the identity of the metabolite will be key for the future interpretation of *in vivo* imaging performed with [^18^F]FSPG or [^18^F]FRPG; the incorporation of the prosthetically labeled glutamate analogs into GSH precursors is an exciting possibility. Other metabolic transformations leading to a more lipophilic metabolite include *N*-acetylation to an *N*-acetyl glutamate derivative, deamination to a glutaric acid derivative and cyclisation to a 5-oxoproline derivative [67–69]. Investigations to identify the metabolite and its effect on imaging are ongoing in our laboratory.

Finally, we asked whether [^18^F]FRPG can be used to non-invasively image tumor redox status. A dynamic marker of the redox status of cells is of great value as it has the potential to inform on tumor progression, response and resistance to therapy. Previously, we showed that oxidative stress reduced intracellular cystine as a result of elevated GSH synthesis, which could be monitored by [^18^F]FSPG [35]. Here, TBHP-induced oxidative stress in ovarian A2780 cells resulted in a similar ~50% reduction in [^18^F]FRPG retention compared to vehicle controls, with antioxidant treatment (NAC) also doubling tumor [^18^F]FRPG retention. These data show [^18^F]FRPG to be a sensitive marker of tumor redox status. *In vivo*, [^18^F]FRPG retention was reduced by 15 % in subcutaneous A2780 tumors following Doxil-induced oxidative stress (Figure 8), with [^18^F]FSPG tumor retention reduced by 42% under the same treatment conditions [35]. The lower dynamic range of response with [^18^F]FRPG is likely a consequence of reduced radiotracer affinity for system x_C_^-^ and may have important implications for treatment response monitoring. Previous findings showed there were no changes in tumor xCT protein levels following 24h treatment with Doxil [35]. Temporal differences in [^18^F]FRPG tumor retention following therapy and the magnitude of change, however, still need to be determined in this model and with other tumor types and treatments.

In conclusion, we have reported a radiolabeled non-natural amino acid, [^18^F]FRPG, which is transported into cells through system x_C_^-^, a key transporter for the maintenance of redox homeostasis. [^18^F]FRPG retains many of the valuable properties of its stereoisomer [^18^F]FSPG with excellent tumor-to-background ratios, equal to, or outperforming [^18^F]FSPG in the range of cancer models tested here. Moreover, the improved metabolic profile of [^18^F]FRPG compared to [^18^F]FSPG in tumors may simplify the interpretation of the resulting images. Non-invasive molecular imaging with [^18^F]FRPG also holds the potential for the monitoring of therapy-induced oxidative stress. Future work will determine the mechanisms governing [^18^F]FRPG metabolism and assess its impact on the non-invasive imaging of system x_C_^-^.

## Abbreviations

[^18^F]FRPG: (*R*)-4-(3-^18^F-fluoropropyl)-L-glutamate
[^18^F]FSPG: (*S*)-4-(3-^18^F-fluoropropyl)-L-glutamate
[^18^F]FDG: [^18^F]2-fluoro-2-deoxy-D-glucose
[^18^F]FASu: ^18^F-5-fluoro-aminosuberic acid
4-FGln: ^18^F-labeled 4-fluoro-glutamine
[^18^F]FDOPA: 6-^18^F-fluoro-3,4-dihydoxy-L-phenylalanine
ANOVA: Analysis of variance
EAATs: excitatory amino acid transporters
EAATi: L-trans-pyrrolidine-2,4-dicarboxylic acid;
GSH: glutathione
LPS: lipopolysaccharide
MIPs: maximum-intensity projections
NAC: N-acetylcysteine
NRF2: nuclear factor erythroid 2-related factor 2
[^18^F]FET: O-(2-^18^F-fluoroethyl)-L-tyrosine
OPA: *ortho*-phthalaldehyde
CPG: p-carboxyphenylglycine
PET: positron emission tomography
p.i.: post injection
ROS: reactive oxygen species
SD: standard deviation
TBHP: tert-butyl hydroperoxide

## Supplementary Material

Supplemental Information - Synthesis and Spectra

Supplemental methods, figures and tables

## Figures and Tables

**Figure:**
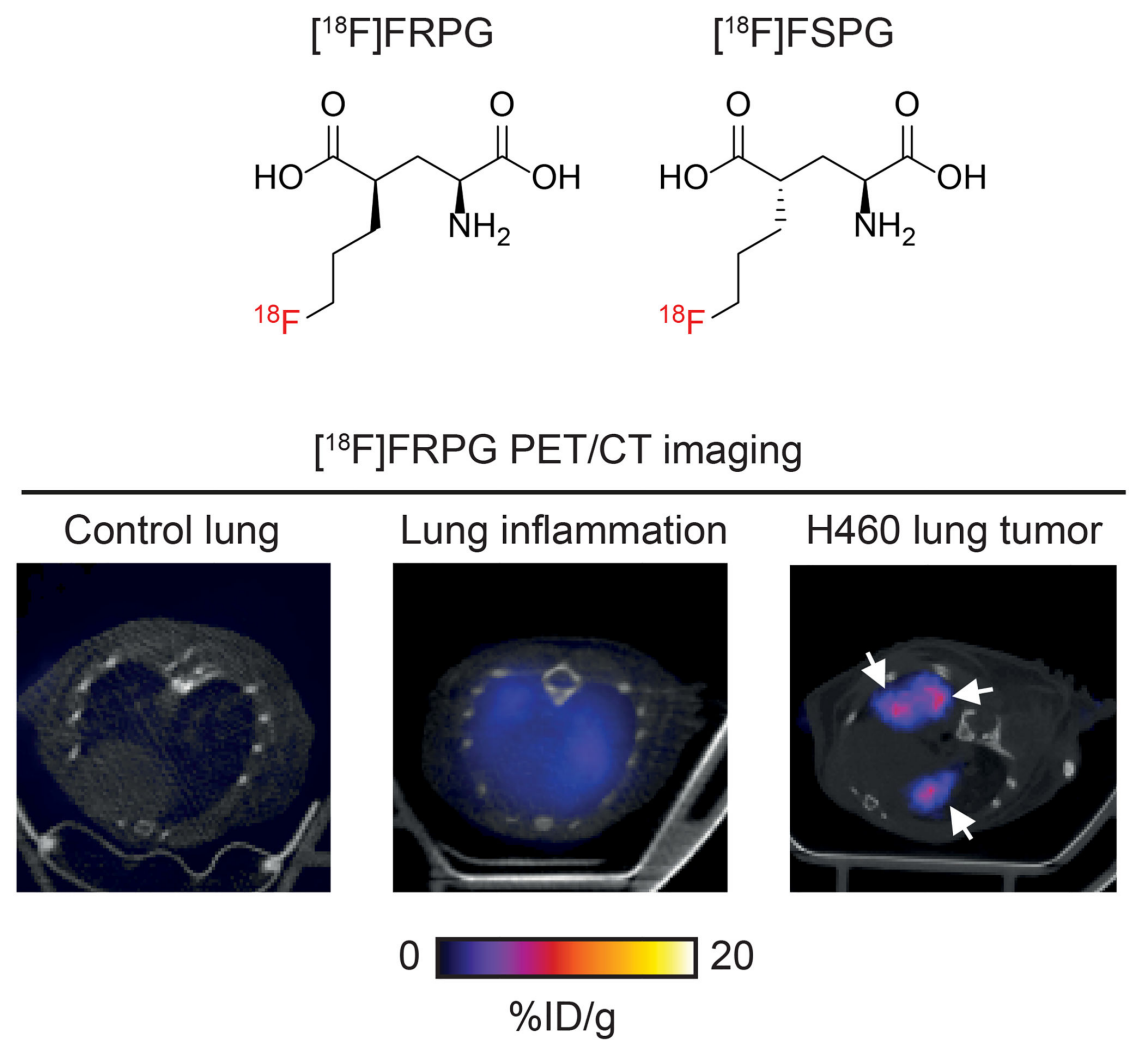
Imaging system x_C_^-^ activity with [^18^F]FRPG PET. [^18^F]FRPG is a glutamate analogue and stereoisomer of the clinically-tested radiotracer, [^18^F]FSPG. [^18^F]FRPG PET/CT imaging revealed excellent tumor retention in small lung lesions, 2.5-fold higher than inflamed lung tissue.
