# Supplementary material for: Radiotracer stereochemistry affects substrate affinity and kinetics for improved imaging of system xC- in tumors
Source: Theranostics. Author manuscript; Available in PMC 2022 Nov 24. (PMC8825600; doi:10.7150/thno.63237)
Supplement: Supplemental methods, figures and tables [file EMS143766-supplement-Supplemental_methods__figures_and_tables.docx]

**­­** SUPPLEMENTARY INFORMATION:

SUPPLEMENTAL METHODS, FIGURES AND TABLES

**Radiotracer stereochemistry affects substrate affinity and kinetics for improved imaging of system x_c_^-^ in tumors**

Hannah E. Greenwood*, Richard Edwards*, Norman Koglin, Mathias Berndt, Friedrich Baark, Jana Kim, George Firth, Eman Khalil, Andre Mueller, Timothy H. Witney

# **Supplementary Methods**

xCT small inhibitory RNA (siRNA)

H460 cells (1 × 10^6^/mL) were seeded in 6-well plates in antibiotic-free RPMI (2 mL) the day before siRNA transfection with lipofectamine 2000 (ThermoFisher) according to the manufacturer’s instructions. Specific siRNAs targeting xCT were custom-made by Thermo Scientific using the following sequences: GGAGUUAUGCAGCUAAUUAtt (sense) and UAAUAAGCUGCAUAACUCCag (antisense), sequences that have been validated previously [1]. Scrambled siRNA was used as a control (Silencer™ Negative Control; Invitrogen). siRNA was added at a final concentration of 25 nM, and xCT knockdown was examined by western blot 48h after transfection.

Bioluminescence imaging

Orthotopic NCI-H460 FLuc tumors were monitored through BLI imaging using an IVIS Spectrum *in vivo* imaging system (PerkinElmer). Images were acquired 4 h post cell inoculation to confirm successful delivery of cells to the lung. Mice were subsequently imaged once a week for the first 3 weeks and then every 3-4 days thereafter. For BLI imaging, mice were anesthetized with isoflurane (1-2 % in O_2_) and injected i.p. with 150 mg/kg firefly luciferin (Promega) before being transferred to the IVIS Spectrum camera and maintained at 37°C. Images were acquired until the luminescent signal plateaued ~20 min p.i. of luciferin, ensuring maximum tumor signal was reached (exposure time 1-60 s, binning 2-8, FOV 23 cm, f/stop 1, no filter). Tumor growth was monitored over 5 weeks or until experimental endpoint. For signal quantification, images were analyzed using Living Image software (PerkinElmer). A region of interest was drawn around the entire thorax and total photon flux was measured (photon/sec). Once the bioluminescent signal reached ~5 × 10^7^ photons/s/cm^3^, mice were selected for PET/CT imaging.

Histology

All lungs where fixed in 10% buffered formalin, 4°C overnight before being stored in 70% ethanol until processing by UCL IQPath (London, UK) for histologic analysis. Formalin fixed paraffin embedded tissues were sliced and stained with hematoxylin & eosin (H&E). Immunohistochemistry (IHC) was performed with a Discovery XT system (Ventana Medical Systems) using the DAB Map detection kit (Ventana). Sections were additionally stained for T-cells (anti-CD3).

*In vivo* metabolite analysis

*In vivo* metabolism of [^18^F]FRPG and [^18^F]FSPG was performed by radio-HPLC analysis. The amount of metabolite and parent tracer were quantified based on the area under the curve (region of interest) for [^18^F]FRPG (retention time 6:51 minutes:seconds) or [^18^F]FSPG (retention time 7:10 minutes:seconds) and their corresponding metabolites ([^18^F]FRPG metabolite retention time 15:15 minutes:seconds, [^18^F]FSPG metabolite retention time 16:30 minutes:seconds), and expressed as a percentage (mean ± SD). Tumor, blood, liver, pancreas, and urine samples were analyzed at 60 min post-injection of the tracer.

A549 cells (5 × 10^6^) in PBS were injected into the flank of female Balb/c nu/nu mice. When the tumors reached ~100 mm^3^ mice were anesthetized with isoflurane (1.5-2% in oxygen) and injected with ~10 MBq of radiotracer through a tail vein cannular. Mice were maintained at 37°C under anesthesia throughout radiotracer uptake. At 60 minutes p.i., the mice were sacrificed by exsanguination via cardiac puncture under terminal anesthesia. Liver, tumor, pancreas, urine, and blood samples were harvested and placed on ice prior to processing. The blood samples were centrifuged (2,000 × *g* for 5 min, 4°C), the plasma was removed and transferred to a 1.5 mL Eppendorf. Ice-cold MeOH (1 mL) was added to the plasma and urine samples, and both were briefly mixed on a Vortex. On ice, ice-cold MeOH (1 mL) was added to the liver, tumor and pancreas samples prior to homogenization using a PRECELLYS® 24 tissue homogenizer. All samples were then centrifuged (12,000 × *g* for 5 min, 4°C) and the supernatant transferred to a glass vial through a Millex 0.2 µm filter (Millipore, Billerica, MA, USA). The samples were diluted with 3 mL mobile phase (H_2_O, 0.1% TFA), passed through another Millex 0.2 µm filter (Millipore, Billerica, MA, USA) and monitored by reverse phase HPLC (2.5 mL injection). Column: ZORBAX ® StableBond 300 C18, 9.4 x 250 mm, 5 µm HPLC column at room temperature; solvent A: H_2_O (0.1% TFA), solvent B: MeOH (0.1% TFA); flow rate: 4 mL/min; UV detector: 254 nm; gradient: 3% B, 0-1 min; 3-5% B, 1-11 min; 5-95% B, 11-16 min; 95-3% B, 16-20 min.

*In vivo* imaging with [^18^F]FRPG following inhibition of system x_c_^-^

3 × 10^6^ H460 cancer cells in 100 µL PBS were injected subcutaneously into female Balb/C nu/nu mice aged 6-9 weeks (Charles River Laboratories). Tumor growth was monitored and [^18^F]FRPG PET/CT imaging was performed as described above once the tumor size reached ~100 mm^3^. Immediately after PET/CT imaging, mice were recovered and randomized. 24 h later, mice where anaesthetized with isoflurane (2-2.5% in O_2_) and received a single 10 µL intratumoral injection of 2.5 mg/kg imidazole ketone erastin (IKE) which was dissolved in Hank Balancing Salt Solution (HBSS) with 5% DMSO. One hour following the administration of IKE, mice were re-imaged by [^18^F]FRPG PET/CT for an hour. Mice remained anaesthetized throughout the IKE treatment and imaging.

# **Supplementary Figures**


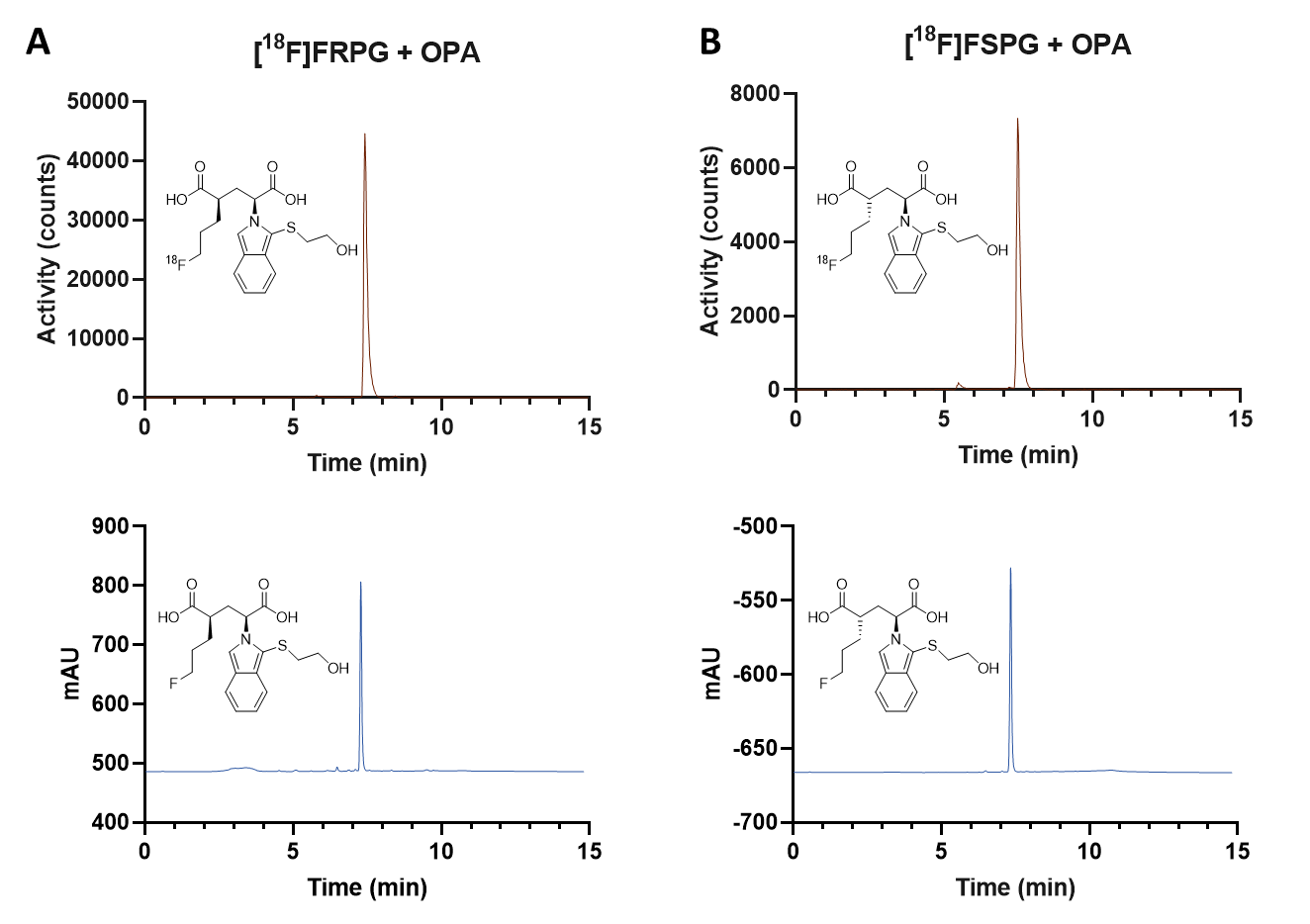


**Supplementary Fig. S1.** Example radio and UV chromatograms of [^18^F]FRPG and [^18^F]FSPG after reaction with OPA. A. Radio-chromatogram of [^18^F]FRPG after reaction with OPA reagent (top) and the corresponding UV-chromatogram (bottom) showing co-elution of the ‘cold’ [^19^F]FRPG-OPA product. B. Radio-chromatogram of [^18^F]FSPG after reaction with OPA reagent (top) and the corresponding UV-chromatogram (bottom) showing co-elution of the ‘cold’ [^19^F]FSPG-OPA product. Column: Chromolith C18 (100 × 4.6 mm); solvent A: H_2_O (0.1% TFA), solvent B: MeOH (0.1% TFA); flow rate: 3 mL/min; UV detector: 314 nm; gradient: 10-90% B, 0-10 min; 90% B, 10-15 min.


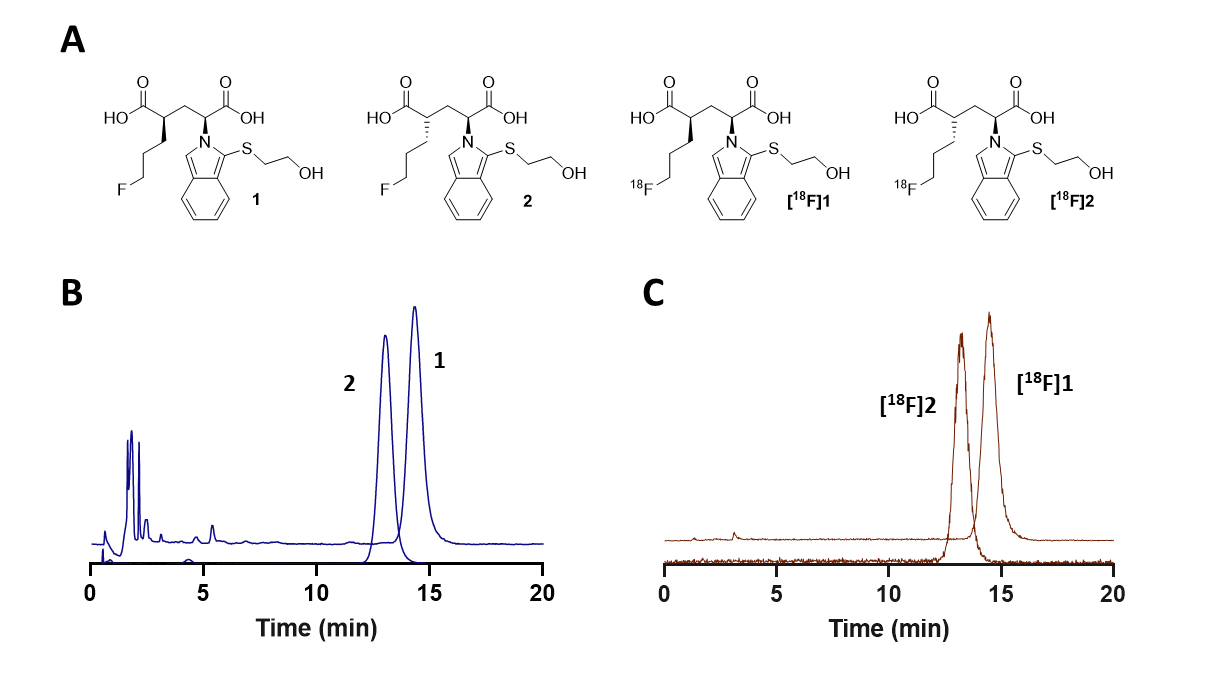


**Supplementary Fig. S2.** Example radio and UV chromatograms of [^18^F]FRPG and [^18^F]FSPG after reaction with OPA, using a 37% isocratic gradient to achieve separation of the two OPA-adducts. A. Structures of the OPA adducts produced upon OPA derivatization of [^18^F]FRPG, [^18^F]FSPG and their corresponding cold reference standards (FRPG-OPA (**1**), FSPG-OPA (**2**), [^18^F]FRPG-OPA ([^18^F]**1**), [^18^F]FSPG-OPA ([^18^F]**2**)). B. UV-chromatograms of [^19^F]FRPG and [^19^F]FSPG after reaction with OPA reagent. C. Radio-chromatograms of [^18^F]FRPG and [^18^F]FSPG after reaction with OPA reagent. Column: Chromolith C18 (100 x 4.6 mm); solvent A: H2O (0.1% TFA), solvent B: MeOH (0.1% TFA); flow rate: 3 mL/min; UV detector: 314 nm; gradient: 5-37 % B, 0-1 min; 37% B, 1-20 min.


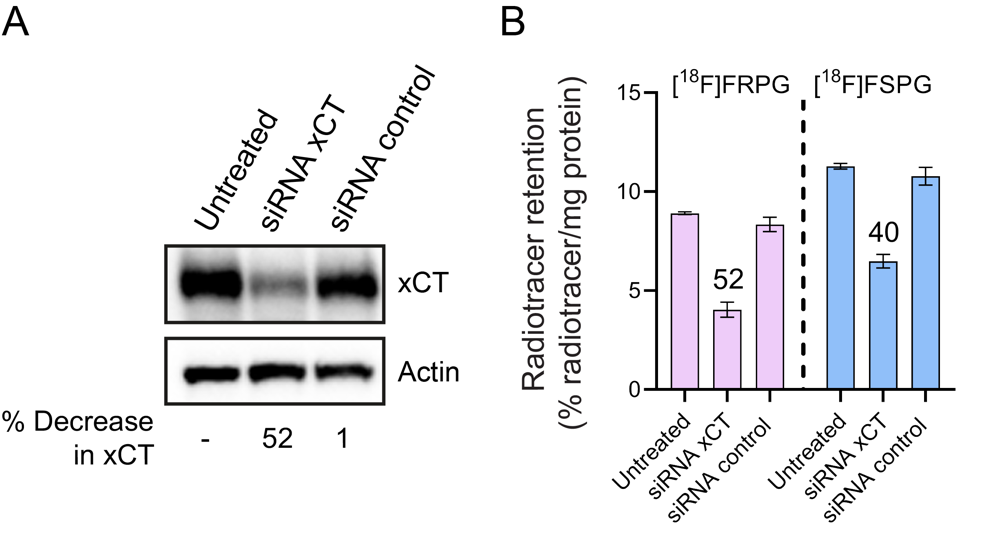


**Supplemental Fig. S3**. Genetic knockdown of xCT reduces tumor retention of [^18^F]FRPG and [^18^F]FSPG. A. xCT protein expression in untreated H460 cells, and cells treated with either xCT siRNA or scrambled control siRNA. Actin was used as a loading control. B. [^18^F]FRPG and [^18^F]FSPG cell uptake following knock-down of xCT expression by siRNA. Data represents 3 technical repeats. The percentage decrease in radiotracer cell retention for xCT siRNA vs. control siRNA treatments are provided.


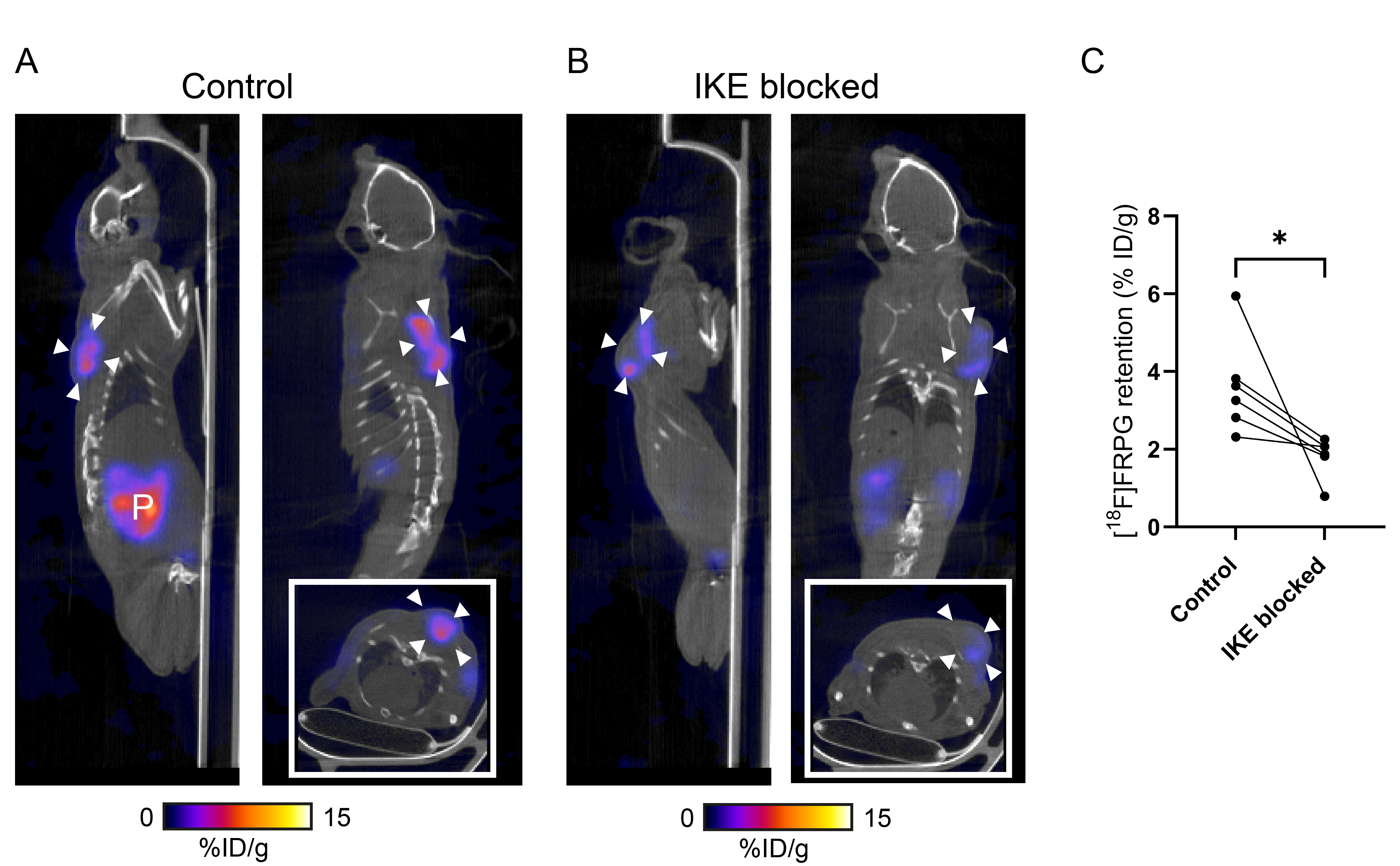


**Supplementary Fig. S4**. The xCT inhibitor IKE reduces [^18^F]FRPG tumor retention. A. Representative single slice sagittal, coronal and axial (inset) PET/CT images in H460 subcutaneous tumor-bearing mouse 40-60 min after injection of ~3 MBq [^18^F]FRPG. B. Representative single slice sagittal, coronal and axial (inset) [^18^F]FRPG PET/CT (summed 40-60 min) images from the same mouse 1 h following the intratumoral injection of IKE. Imaging of control mice and IKE treated mice was performed 24 h apart in the same animals. White arrowheads indicate the tumor margins. P, pancreas. C. Quantification of radiotracer retention in matched H460 tumors imaged 24 h apart. Data points represent individual animals. *, *P* < 0.05.

 **Supplementary Fig. S5.** Representative radio-HPLC chromatograms and *in vivo* metabolite analysis of [^18^F]FRPG from tumor, blood, liver, pancreas, and urine samples.

**Supplementary Fig. S6.** Representative radio-HPLC chromatograms and *in vivo* metabolite analysis of [^18^F]FSPG from tumor, blood, liver, pancreas, and urine samples.


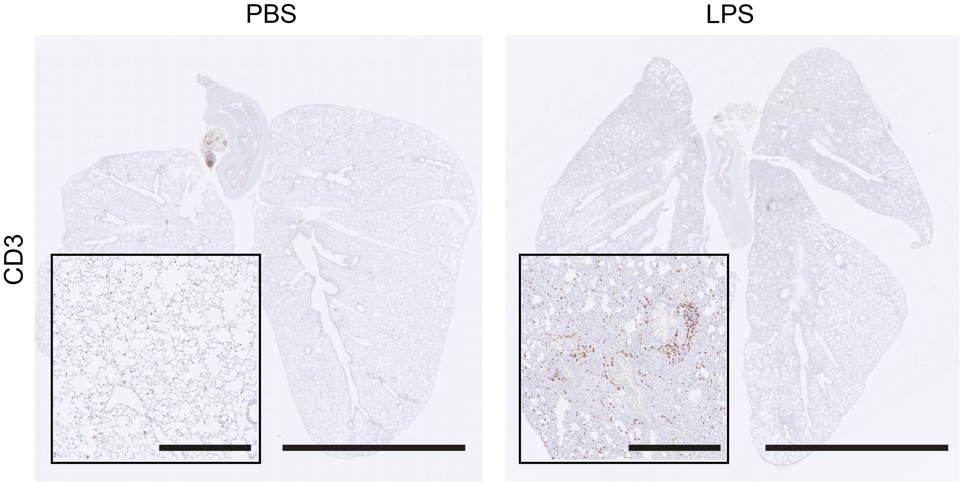


**Supplementary Fig. S7**. Representative CD3 IHC staining of lung sections, showing increased T-cell infiltration with LPS treatment compared to PBS-treated control lungs. Main images are shown at 0.46× magnification (scale bars, 5 mm) and inserts at 5× magnification (scale bars, 500 µm).


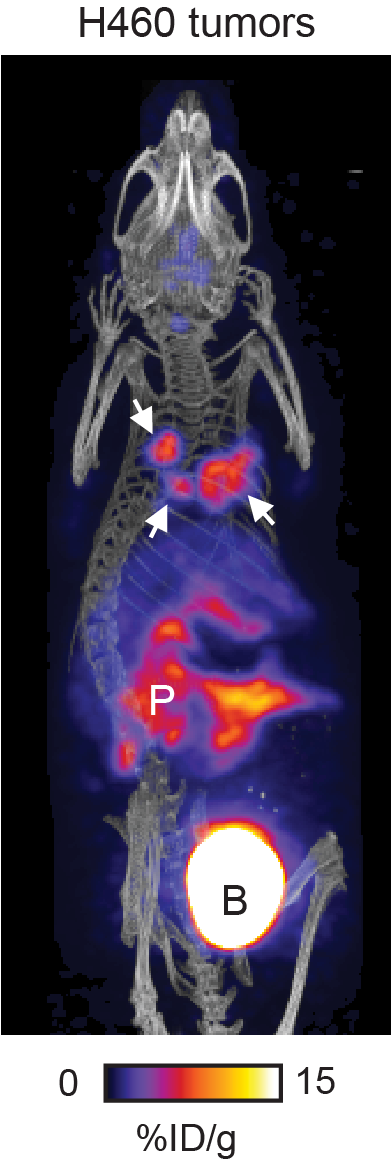


**Supplementary Fig. S8.** [^18^F]FRPG PET/CT maximum intensity projection 40-60 min p.i. of an orthotopic H460 lung tumor-bearing mouse. White arrows indicate the presence of multiple tumor lesions. P, pancreas; B, bladder.

**Supplementary Tables**

**Supplementary Table S1.** Percentage of intact parent radiotracer ([^18^F]FRPG or [^18^F]FSPG) and corresponding metabolite in tissue samples, as determined by radioHPLC.

| Tissue | Percentage radioactivity: [^18^F]FRPG | | Percentage radioactivity: [^18^F]FSPG | |
| --- | --- | --- | --- | --- |
|  | Parent | Metabolite | Parent | Metabolite |
| Tumor | 97.5 ± 0.5 | 2.6 ± 0.5 | 84.5 ± 7.4 | 15.5 ± 7.4 (*) |
| Blood | 48.0 ± 4.4 | 52.0 ± 4.4 | 69.1 ± 3.4 | 30.9 ± 3.4 (***) |
| Urine | 69.3 ± 4.2 | 30.7 ± 4.2 | 87.0 ± 1.4 | 13.0 ± 1.4 (**) |
| Liver | 93.3 ± 2.07 | 6.7 ± 2.07 | >97 | <3 (*) ^#^ |
| Pancreas | 91.0 ± 1.0 | 9.0 ± 1.0 | 96.7 ± 1.3 | 3.3 ± 1.3 (**) |

^#^, metabolite below detection threshold. *, *P* < 0.05; **, *P* < 0.01; ***, *P* < 0.001. Data are presented as mean ± SD

(*n* = 3-5).

**References**

1. Beinat C, Gowrishankar G, Shen B, Alam IS, Robinson E, Haywood T, et al. The Characterization of (18)F-hGTS13 for Molecular Imaging of xC- Transporter Activity with PET. Journal of nuclear medicine : official publication, Society of Nuclear Medicine. 2019; 60: 1812-7.
