# Supplementary material for: Radiotracer stereochemistry affects substrate affinity and kinetics for improved imaging of system xC- in tumors
Source: Theranostics. 2022 Jan 24;12(4):1921–36. doi: 10.7150/thno.63237 (PMC8825600; doi:10.7150/thno.63237)
Supplement: Supplementary file 2 [file thnov12p1921s2.pdf]

SUPPLEMENTARY INFORMATION:

SYNTHESIS AND SPECTRA

Radiotracer stereochemistry affects substrate affinity and kinetics for improved imaging of system x_c^- in tumors

Hannah E. Greenwood*, Richard Edwards*, Norman Koglin, Mathias Berndt, Friedrich Baark, Jana Kim, George Firth, Eman Khalil, Andre Mueller, Timothy H. Witney

Synthesis of [19F]FRPG cold standard

[19F]FRPG 'cold' standard was obtained from Life Molecular Imaging. Procedures for the synthesis of [19F]FRPG were obtained from the company and are reported in short below.

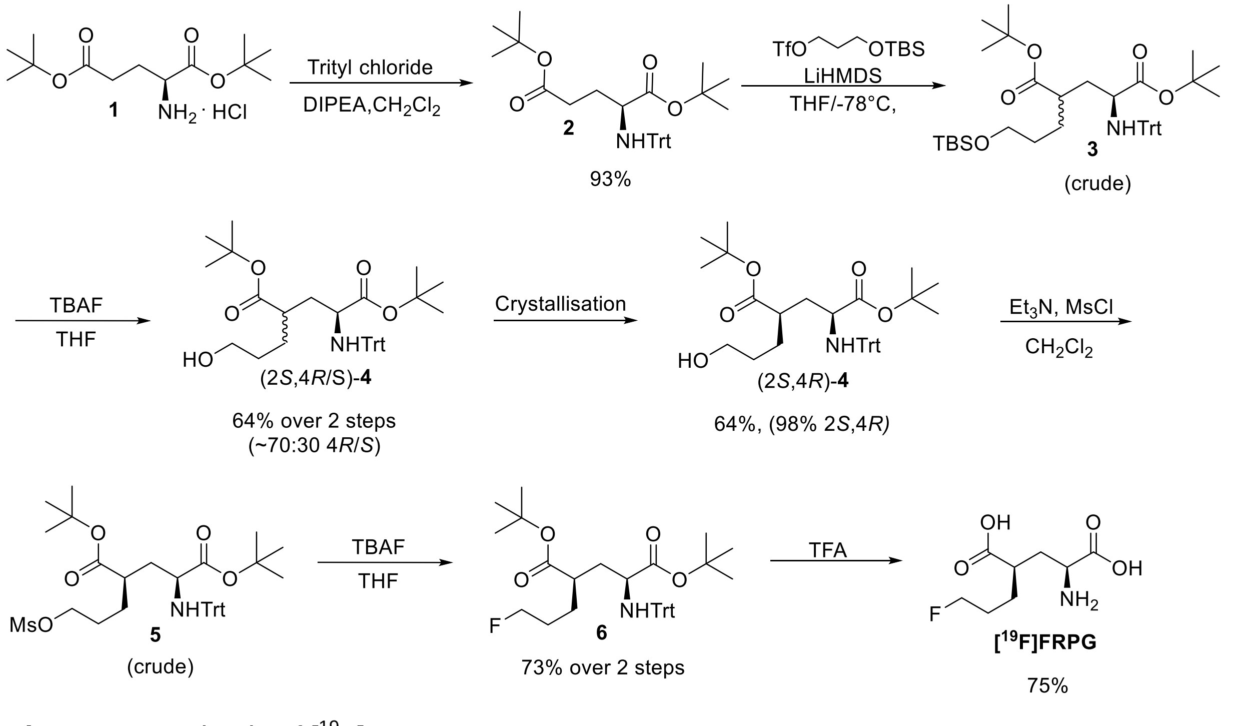

Scheme 1. Synthesis of [19F]FRPG.

Di-tert-butyl-(2*S*)-2-(tritylamino)pentane dioate (**2**)

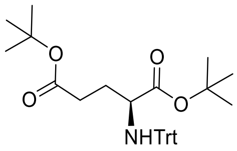

To a solution of di-*tert*-butyl ʟ-glutamate hydrochloride (**1**) (400 g, 1.35 mol) in dichloromethane (2.0 L) was added a solution of DIPEA (402 g, 3.11 mol) in dichloromethane (1.0 L). The resulting solution was cooled to 0 to 5 ^{o}C and triphenylmethyl chloride (339 g, 1.22 mmol) was added portion wise. The reaction solution was stirred at room temperature for 12 h. After the reaction was complete, the reaction mixture was quenched with 15% citric acid solution (1.6 L) and stirred for 15 min. The organic solution was separated, washed with water (2.8 L), followed by 5% sodium bicarbonate solution (1.0 L), and then 10% brine solution (1.0 L). Finally, the organic layer was dried over anhydrous sodium sulfate (50 g), and concentrated *in vacuo* to give (*S*)-di-tert-butyl 2-(tritylamino)pentane dioate (**2**) (633 g, 94%) as a solid.

^{1}H NMR (400 MHz, $CDCl_3$) δ 7.51 (br. d, 6H) 7.24-7.29 (m, 6H), 7.16-7.21 (m, 3H), 3.37 (br. s, 1H), 2.76 (br. d, 1H), 2.51 (ddd, 1H), 1.90-1.20 (m, 3H), 1.47 (s, 9H), 1.17 (s, 9H).

Di-tert-butyl (2*S*)-4-(3-hydroxypropyl) 2-(tritylamino)pentane dioate ((2*S*,4*R*/*S*)-**4**)

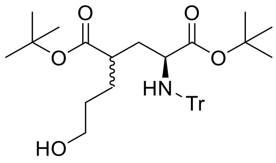

To a stirred solution of (*S*)-di-tert-butyl-2-(tritylamino)pentane dioate (350 g, 0.697 mol) in THF (4.55 L) was added LiHMDS (4.19 L, 4.19 mol, 1.0 M in THF) slowly at -78 °C and stirred for 1 h. To this solution was slowly added a solution of 3-(*tert*-butyl dimethylsilyloxy)propyl trifluoromethanesulfonate (675 g, 2.1 mol) in DCM (4.5 L). The resulting solution was stirred for 2 hrs. After the completion of the reaction, the reaction mixture was quenched with 2 N NH_4Cl solution (3.5 L). The layers were separated, and the aqueous layer was extracted with ethyl acetate (2 x 2.28 L). The combined organic layers were washed with 20% brine (3.5 L) and dried over anhydrous Na_2SO_4 (105 g) and concentrated under reduced pressure to obtain the crude compound as a yellowish oil. The crude compound was purified by column chromatography on silica gel (100 – 200 mesh) using 0 to 5% ethyl acetate in hexane to give

412 g of the TBS protected alcohol ((2S,4*R*/*S*)-**3**) (mixture of diastereomers (2*S*,4*R*/*S*) as a colourless oil.

To a solution of the partially purified TBS protected alcohol ((2S,4*R*/*S*)-**3**) (410 g crude, 0.608 mol) in THF (1.64 L) was added a solution of TBAF·3H_2O (383 g, 1.22 mol) in THF (2.46 L) at 0 °C slowly. The reaction mixture was allowed to warm to RT and stirred at this temperature for 3 hrs. After the reaction was complete, the reaction mixture was quenched with deionized water (4.1 L) and stirred for 30 min. The layers were separated, and the aqueous portion was extracted with ethyl acetate (2 X 1.5 L). The combined organic layers were dried over anhydrous Na_2SO_4 (100 g) and concentrated under reduced pressure to obtain the crude compound. The crude was purified by column chromatography on silicagel (60 – 120 mesh) using 10 to 30% ethyl acetate in hexane as eluant to obtain 250 g of the alcohol product ((2*S*,4*R*/*S*)-**4**) as a liquid (64%, over 2 steps). The product was present as a ~70:30%, (2*S*,4*R*):(2*S*,4*S*) diastereomeric mixture.

^{1}H NMR (400 MHz, $CDCl_3$) δ 7.45-7.53 (m, 6H) 7.21-7.30 (m, 6H), 7.12-7.20 (m, 3H), 3.55-3.67 (m, 2H), 3.23-3.34 (m, 1H), 2.70-2.82 (m, 1H), 2.35-2.45 (m, 1H), 2.06-2.20 (m, 1H), 1.48-1.78 (m, 5H), 1.47 (s, 9H), 1.16 (s, 9H).

MS (ESI) $C_{35}H_{45}NO_5$: m/z 560 $[M]^+$.

<u>Crystallisation</u>

Purification-I:

Column purified (2*S*,4*R*/*S*)-**4** (250 g) was dissolved in n-hexane (725 mL) and stirred at 25-30 °C for 30 minutes. The resulting solution was cooled to 0 to 5 °C for 30 minutes and then gradually allowed to warm to 25-30 °C and maintained at this temperature for 2 hours. A pale yellow solid precipitated in solution was stirred at 25-30 °C for an additional hour. The solid was filtered using a buckner funnel and washed with n-hexane (500 mL) before being dried under vacuum for an hour to give 150.5 g (**4**) as a pale yellow solid. Chiral purity = 94.10% (2*S*,4*R*) : 5.90% (2*S*,4*S*), Achiral purity = 99%.

Purification-II:

The above purified material (150.5 g), was dissolved in n-hexane (375 mL) and heated to 35 to 40 °C and maintained at this temperature for 2 hours. The resulting slurry was filtered using a buckner funnel and the collected solid was washed with n-hexane (75 mL) and dried under

vacuum for one hour to give 112.5 g (2*S*,4*R*)-**4** as a white solid. Chiral purity = 98% (2*S*, 4*R*): 2.0% (2*S*, 4*S*), Achiral purity > 99%.

(4*R*)-4-(3-fluoropropyl)-L-glutamic acid ([^{19}F]FRPG)

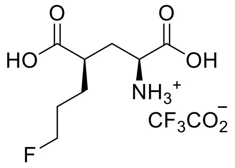

To a solution of (2*S*,4*R*)-**4** (50 g, 89 mmol) in dichloromethane (400 mL) at 0°C was added triethylamine (13.6 g, 134 mmol) and stirred for 10 min. To the above solution was added a solution of mesyl chloride (8.3 mL, 107 mmol) in dichloromethane (100 mL) slowly. The reaction mixture was stirred for 3 h. An additional portion of triethylamine (4.5 g, 0.0445 mol) and mesyl chloride (3.05 g, 26.7 mol) was added and stirred for an additional 1h. After the reaction was complete, the reaction mixture was quenched with deionised water (500 mL). The organic layer was extracted with dichloromethane (250 mL). The combined organic layers were washed with 20% brine (250 mL) and dried over anhydrous Na_2SO_4 (5 g) and concentrated under vacuum to obtain the crude mesylate (**5**) (66 g). The crude compound obtained was taken for the next step without further purification.

To a solution of **5** (65.27 g) in THF (653 mL) at 30 ^{o}C was added a 1.0 M solution of TBAF in THF (716 mL, 716 mmol) in one portion. Resulting solution was refluxed for 1 h. After the reaction was complete, the reaction mixture was concentrated under reduced pressure to obtain the crude compound. To the crude was added DCM (350 mL) and deionised water (326 mL). The organic layer was separated and washed with 10% brine (326 mL), dried over anhydrous Na_2SO_4 and concentrated under vacuum to obtain the crude compound (**6**) (51 g). To the crude compound (51 g) was added n-hexane (55 mL) and the mixture was stirred overnight. The solid obtained was filtered and washed with n-hexane (102 mL). The compound was dried under vacuum for an hour to obtain pure **6** (36.5 g).

To **6** (45 g) at 0 to 5 °C was added trifluoro acetic acid (174 g) drop wise over 1 hour. After addition was complete, the reaction was maintained at the same temperature for 30 minutes and then gradually allowed to warm to 25-30 °C. The reaction mixture was maintained at the same temperature for 3 days. The reaction was monitored by TLC and after the reaction was complete, the volatiles were removed under reduced pressure. The resulting mixture was

triturated with diethyl ether (2 x 225 mL) and concentrated. The crude obtained was stirred in diethyl ether (225 mL) overnight. A fluffy solid was formed and isolated on a buckner funnel, washed with diethyl ether and then dried under suction for 10 minutes. The solid was dried under vacuum for 2 hours to obtain crude [^{19}F]FRPG (Crude, 18.5 g).

To a stirred solution of crude [^{19}F]FRPG (18.5 g) in DM water (450 mL) was added 1 N sodium hydroxide solution (102 mL) in order to adjust the pH to 7.4. After adjusting the pH, the resulting solution was concentrated at 60 to 65 °C under reduced pressure for 3 hours. The solid obtained was triturated with methanol (90 mL) and toluene (135 mL). The solid obtained was purified using prep. HPLC. The purified material was concentrated at 50 to 55 °C under reduced pressure and the material was triturated with methanol (90 mL) followed by toluene (135 mL) to obtain pure [^{19}F]FRPG trifluoroacetic acid salt (12.5 g, 75%) as a white solid.

^{1}H NMR (400 MHz, D_2O) δ 4.61 (t, *J* = 5.9 Hz, 1H), 4.52 – 4.46 (m, 1H), 3.74 (dd, *J* = 9.1, 4.1 Hz, 1H), 2.44 (dddd, *J* = 9.9, 7.9, 5.7, 4.5 Hz, 1H), 2.19 (ddd, *J* = 14.9, 9.7, 4.1 Hz, 1H), 1.93 (ddd, *J* = 14.9, 9.1, 4.5 Hz, 1H), 1.81 – 1.57 (m, 4H).

^{13}C NMR (101 MHz, D_2O) δ 182.95, 174.52, 120.69, 117.79, 114.89, 85.87, 84.30, 28.13, 28.08, 27.78, 27.59.

^{19}F NMR (376 MHz, D_2O) δ -75.56, -216.90.

MS (ESI); m/z =208 $[M+H]^+$

The data are in agreement with those previously reported for the 2*S*,4*S* isomer [1]. Importantly, the compound is stable for > 1 year at -20 °C, as measured by ^{1}H, ^{19}F and ^{13}C NMR.

Synthesis of [^{19}F]FSPG cold standard

[^{19}F]FSPG ‘cold’ standard was obtained from Life Molecular Imaging. Procedures for their synthesis can be found in patents published by the company and are reported in short below [2,3].

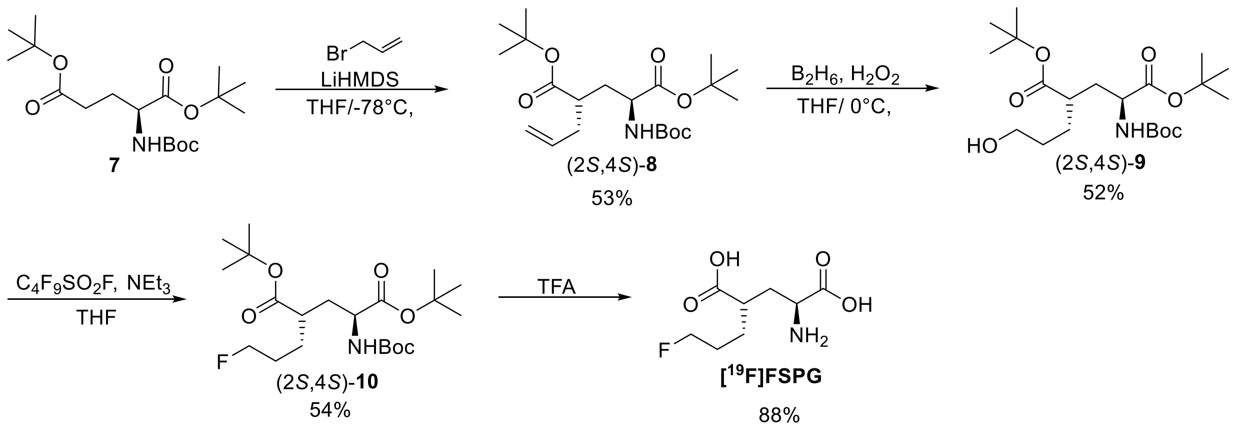

Scheme 2. Synthesis of [^{19}F]FSPG.

<u>Di-tert-butyl (2*S*,4*S*)-4-allyl-2-((tert-butoxycarbonyl)amino)pentane dioate ((2*S*,4*S*)-**8**)</u>

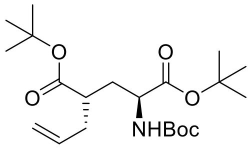

Di-tert-butyl Boc-glutamate [4] (**7**) (26.96 g, 75 mmol) was dissolved in THF (220 mL) and cooled to -78° C before addition of LiHMDS (165 mL, 165 mmol, 1M in THF) dropwise over a period of two hours at this temperature. The mixture was stirred at -78° C for another 2 hours. Allyl bromide (27.22 g, 225 mmol) was then added dropwise, and after 2 h at this temperature, the cooling bath was removed and 2N aqueous hydrochloric acid (375 mL) and ethyl acetate (1.25 L) were added. The organic phase was separated off, washed with water until neutral, dried over sodium sulphate, filtered, and the filtrate concentrated under reduced pressure. The crude product was purified by flash chromatography using a hexane/ethyl acetate gradient, and the appropriate fractions were combined and concentrated to give (2*S*,4*S*)-**8** (15.9 g, 53.1%).

^{1}H NMR (300 MHz, $CDCl_3$) δ 5.68-5.77 (m, 1H), 5.02- 5.11 (m, 2H), 4.85-4.92 (d, 1H), 4.10-4.18 (m, 1H), 2.40-2.48 (m, 1H), 2.25-2.39 (m, 2H), 1.81-1.92 (m, 2H), 1.32-1.58 (m, 27H).

MS (ESI): m/z=400 $[M+H]^+$

<u>Di-tert-butyl (2*S*,4*S*)-4-(3-hydroxypropyl)-2-((tert-butoxycarbonyl)amino)pentane dioate ((2*S*,4*S*)-**9**)</u>

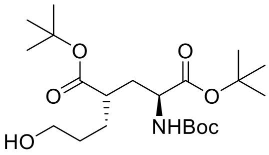

To a solution of (2*S*,4*S*)-**8** (15.58 g, 39 mmol) in THF (200 mL) under N_2 at 0 °C was added diborane/THF complex (54.6 mL, 54.6 mmol, 1M in THF) drop wise over a period of 20 minutes. The mixture was stirred for 2 h at 0 °C and overnight at room temperature. Aqueous sodium hydroxide (58.5 mL, 58.5 mmol, 1 N) was then added dropwise followed by dropwise addition of 30% aqueous hydrogen peroxide solution (58.5 mL) at 0° C. After one hour at this temperature, the reaction mixture was diluted with water, the THF was removed under reduced pressure and the aqueous remainder was extracted with ethyl acetate. The organic layer was separated, washed until neutral with water, dried over sodium sulphate, filtered and the filtrate was evaporated to dryness. The resulting crude product was purified by flash chromatography with hexane/ethyl acetate on silica gel. The product fractions were combined, and the solvents were evaporated to dryness to give (2*S*,4*S*)-**9** (8.5 g, 52.2%).

^{l}H NMR (300 MHz, $CDCl_3$) δ 4.95-5.03 (d, 1H), 4.15-4.22 (m, 1H), 3.58-3.68 (m, 2H), 2.33-2.40 (m, 1H), 2.05-2.12 (m, 1H), 1.73-1.94 (m, 4H), 1.60-1.70 (m, 2H), 1.32-1.58 (m, 27H).

MS (ESI): m/z=418 $[M+H]^+$

<u>Di-tert-butyl (2*S*,4*S*)-4-(3-fluoropropyl)-2-((tert-butoxycarbonyl)amino)pentane dioate ((2*S*,4*S*)-**10**)</u>

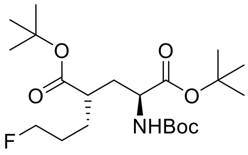

To a solution of (2*S*,4*S*)-**9** (29.22 g, 70 mmol) in THF (700 mL) was added of trimethylamine (42.5 g, 420 mmol). After addition of perfluorobutane sulfonyl fluoride (25.14 mL, 140 mmol) and triethylamine/hydrogen fluoride (22.57 g,140 mmol), the reaction mixture was stirred for 65 h at room temperature. The volatiles were removed under reduced pressure and the resulting crude product was purified by flash chromatography with hexane/ethyl acetate on silica gel. The product fractions were combined and concentrated under vacuum to give (2*S*,4*S*)-**10** (15.9 g, 54.1%).

^{l}H NMR (300 MHz, $CDCl_3$) δ 4.85- 4.90 (d, 1H), 4.48-4.55 (m, 1H), 4.30-4.40 (m, 1H), 4.15-4.22 (m, 1H), 2.33-2.42 (m, 1H), 1.60-1.95 (m, 6H), 1.40-1.55 (m, 27H).

MS (ESI): m/z=420 $[M+H]^+$

<u>(4*S*)-4-(3-fluoropropyl)-ʟ-glutamic acid ([^{19}F]FSPG)</u>

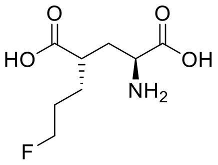

(2*S*,4*S*)-**10** (15.52 g, 37 mmol) was cautiously dissolved in trifluoroacetic acid (110 mL) and stirred for 3 days at room temperature. The reaction mixture was then evaporated to dryness and the resulting crude product was redistilled three times with diethyl ether. The residue was dissolved in of water (200 mL) and the pH was adjusted to 2 with addition of 1 N hydrochloric acid (20 mL). The aqueous solution was then washed successively with dichloromethane and ethyl acetate before adjusting the pH to 7.4 with 1 N sodium hydroxide (~65 mL). The solution was freeze-dried and the solid obtained was purified using prep. HPLC. The purified material was concentrated at 50 to 55 °C under reduced pressure to give pure [^{19}F]FSPG (7.5 g, 88%) as a white solid.

^{1}H NMR (400 MHz, D_2O) δ 4.65 – 4.57 (m, 1H), 4.53 – 4.45 (m, 1H), 3.76 (dd, *J* = 8.0, 6.3 Hz, 1H), 2.74 (dq, *J* = 9.2, 6.1 Hz, 1H), 2.14 (ddd, *J* = 14.3, 9.4, 6.3 Hz, 1H), 2.02 (ddd, *J* = 14.3, 8.1, 5.2 Hz, 1H), 1.86 – 1.66 (m, 4H).

^{13}C NMR (101 MHz, D_2O) δ 179.43, 173.84, 85.59, 84.02, 52.99, 41.91, 32.67, 27.80, 27.74, 27.13, 26.94.

^{19}F NMR (376 MHz, D_2O) δ -217.49.

MS (ESI): m/z=208 $[M+H]^+$

The data are in agreement with those previously reported for the 2*S*,4*S* isomer [1]. Importantly, the compound is stable for > 1 year at -20 °C, as measured by ^{1}H, ^{19}F and ^{13}C NMR.

Synthesis of [^{18}F]FRPG and [^{18}F]FSPG Precursors

[^{18}F]FRPG and [^{18}F]FSPG precursors were obtained from Life Molecular Imaging. Procedures for their synthesis can be found in patents published by the company and are reported in short below.[5]

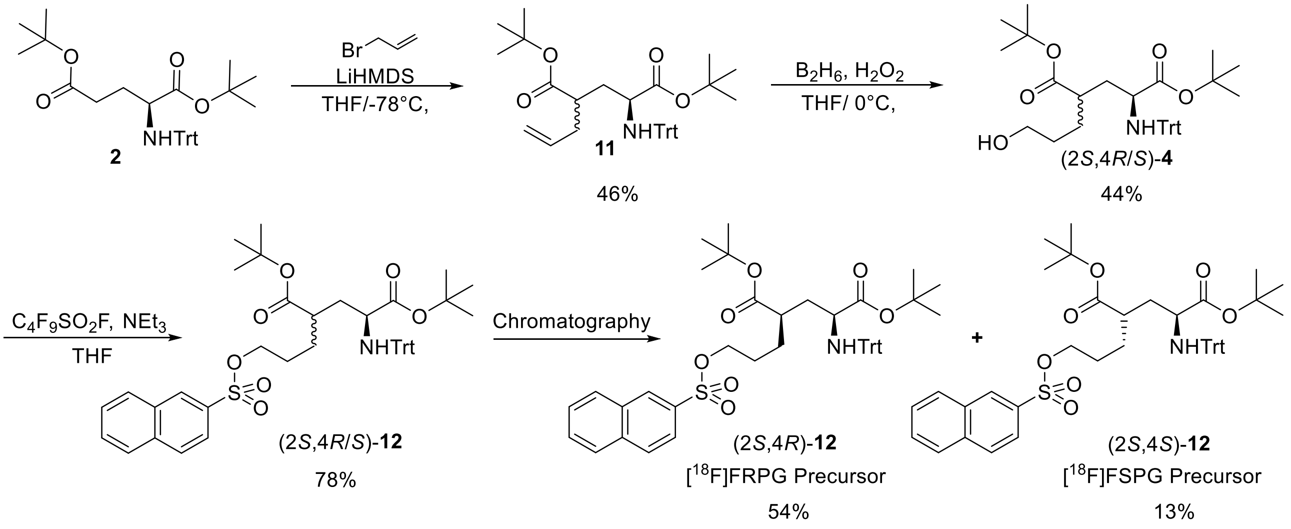

Scheme 3. Synthesis of [^{18}F]FRPG and [^{18}F]FSPG Precursors

<u>Di-tert-butyl (2*S*)-4-allyl-2-(tritylamino)pentanedioate (**11**)</u>

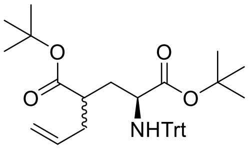

To a solution of di-tert-butyl-(2*S*)-2-(tritylamino)pentanedioate (**2**) (1.99 g, 1.85 mmol) in THF (50 mL) at -78°C was added LiHMDS (47 mL, 47 mmol, 1M in THF) slowly (over a period of 20 min). The solution was stirred for 2 h at -78°C, and allyl bromide (1.44 g, 11.9 mmol) was added drop wise at -78°C. After stirring at this temperature for 1.5 h, the reaction mixture was quenched with a saturated aqueous solution of NH_4Cl, and allowed to warm to room temperature. The volatiles were removed under vacuum and the resulting aqueous solution was extracted with dichloromethane. The combined organic layers were washed with water and dried over sodium sulfate. After filtration, the solution was evaporated and the crude product was purified by flash chromatography (silica, ethyl acetate/n-hexane) to give the di-tert-butyl (2*S*)-4-allyl-2-(tritylamino)pentane dioate (**11**) (1.01 g, 46%) as a mixture of (2*S*,4*S*)/(2*S*,4*R*) diastereoisomers.

^1H NMR (400 MHz, $CDCl_3$) δ 7.45-7.51 (m, 6H), 7.21-7.27 (m, 6H), 7.14-7.18 (m, 3H), 5.63-5.76 (m, 1H), 4.96-5.06 (m, 2H), 3.26-3.33 (m, 1H), 2.74 (br. d, 1H), 2.43-2.51 (m, 1H), 2.10-2.37 (m, 3H), 1.69-1.77 (m, 1H), 1.45 (s, 9H), 1.16 (s, 9H).

MS (ESI) $C_{3}5H_{4}3NO4$: m/z 541 $[M]^+$

<u>Di-tert-butyl (2*S*)-4-(3-hydroxypropyl) 2-(tritylamino)pentane dioate ((2*S*,4*R*/*S*)-**4**)</u>

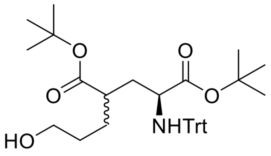

Borane tetrahydrofuran complex (1M, 2.8 mL, 2.8 mmol) was added drop wise to a solution of di-tert-butyl (2*S*)-4-allyl-2-(tritylamino)pentane dioate (**11**) (1.00 g, 1.85 mmol) in THF (10 mL) at 0 °C. The resulting mixture was stirred for 2 h at 0 °C followed by 16 h at room temperature. The solution was then cooled to 0 °C. NaOH (1M, 3 mL) and H_2O_2 (30% in water, 3 mL) were added drop wise. The mixture was stirred at 0 °C for 1 h. Water (5 mL) was added and the mixture was concentrated under reduced pressure. The aqueous residue was extracted with ethyl acetate. The combined organic fractions were washed with brine, dried over sodium sulfate, filtrated and concentrated. The crude product was purified by flash chromatography (silica, 15 ethyl acetate/hexane) to give di-tert-butyl (2*S*)-4-(3-hydroxypropyl) 2-(tritylamino)pentanedioate ((2*S*,4*R*/*S*)-**4**) (0.46 g, 44%) as a mixture of (2*S*,4*S*)/(2S,4*R*) diastereoisomers.

(See above for characterization)

<u>$[^{18}F]$FRPG and $[^{18}F]$FSPG Precursors ((2*S*,4*R*)-**12** and (2*S*,4*S*)-**12**)</u>

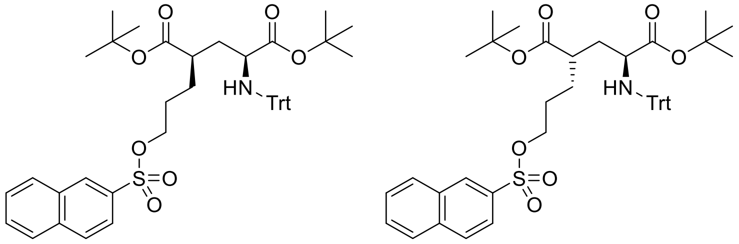

To a solution of di-tert-butyl (2*S*)-4-(3-hydroxypropyl) 2-(tritylamino)pentanedioate ((2*S*,4*R*/*S*)-**4**) (0.457 g, 0.816 mmol) in dichloromethane (10mL) at 0° C, were added triethylamine (0.68 mL, 4.90 mmol) and naphthalene-2-sulfonyl chloride (0.370 g, 1.63 mmol). The resulting mixture was stirred at 0° C for 2 h and for 16 h at room temperature. The solution was concentrated and

the crude product was purified by flash chromatography (silica, ethyl acetate/hexane) to give the naphthylsulfonyl precursors (0.479mg, 78%) as a mixture of (2*S*,4*S*)/(2*S*,4*R*) diastereoisomers. The isomers were separated by chiral HPLC (Chiralpak IC 5 pm 250x30 mm, ethanol/methanol 1:1, 30 mL/min) to give the pure di-tert-butyl (2*S*,4*R*)-4-(3-((naphthalen-2-ylsulfonyl)oxy)propyl)-2-(tritylamino)pentanedioate ((2*S*,4*R*)-**12**) (323 mg, 53%) and di-tert-butyl (2*S*,4*S*)-4-(3-((naphthalen-2-ylsulfonyl)oxy)propyl)-2-(tritylamino)pentanedioate ((2*S*,4*S*)-**12**) (80 mg, 13%) as white solids.

Di-tert-butyl (2*S*,4*R*)-4-(3-((naphthalen-2-ylsulfonyl)oxy)propyl)-2-(tritylamino)pentanedioate ((2*S*,4*R*)-**12**)

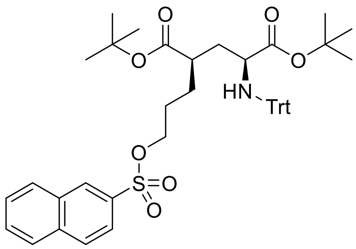

^{1}H NMR (400 MHz, $CDCl_3$) δ 8.50 – 8.46 (m, 1H), 7.98 (dd, *J* = 8.8, 7.9 Hz, 2H), 7.94 – 7.91 (m, 1H), 7.84 (dd, *J* = 8.7, 1.8 Hz, 1H), 7.66 (dddd, *J* = 18.7, 8.2, 7.0, 1.4 Hz, 2H), 7.49 – 7.43 (m, 6H), 7.22 (dd, *J* = 8.3, 6.8 Hz, 6H), 7.18 – 7.12 (m, 3H), 4.02 (t, *J* = 6.4 Hz, 2H), 3.22 (s, 1H), 2.71 (s, 1H), 2.34 (p, *J* = 6.2 Hz, 1H), 2.10 (ddd, *J* = 14.2, 8.3, 6.0 Hz, 1H), 1.65 – 1.55 (m, 3H), 1.50 – 1.42 (m, 2H), 1.40 (s, 9H), 1.13 (s, 9H).

^{13}C NMR (101 MHz, $CDCl_3$) δ 174.50, 173.82, 146.14, 135.29, 132.93, 131.99, 129.69, 129.67, 129.35, 129.34, 128.85, 128.01, 127.94, 127.81, 126.42, 122.54, 80.83, 80.57, 71.42, 70.44, 55.04, 42.07, 37.98, 28.16, 28.11, 27.88, 26.68.

MS (ESI) C45H51NO7S: m/z 750 $[M]^{+}$

Di-tert-butyl (2*S*,4*S*)-4-(3-((naphthalen-2-ylsulfonyl)oxy)propyl)-2-(tritylamino)pentanedioate ((2*S*,4*S*)-**12**)

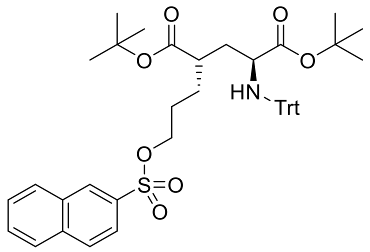

^{1}H NMR (400 MHz, $CDCl_3$) δ 8.48 (d, *J* = 1.9 Hz, 1H), 8.05 – 7.95 (m, 2H), 7.98 – 7.90 (m, 1H), 7.85 (dt, *J* = 8.6, 2.3 Hz, 1H), 7.73 – 7.60 (m, 2H), 7.47 – 7.39 (m, 5H), 7.36 – 7.09 (m, 11H), 4.05 (t, *J* = 6.3 Hz, 2H), 3.20 (s, 1H), 2.75 (s, 1H), 2.06 (ddd, *J* = 14.3, 9.7, 5.0 Hz, 2H), 1.73 – 1.35 (m, 3H), 1.27 (s, 9H), 1.12 (s, 9H).

^{13}C NMR (101 MHz, $CDCl_3$) δ 174.54, 174.09, 147.01, 146.37, 135.43, 133.04, 132.12, 129.84, 129.50, 129.48, 129.00, 128.15, 128.08, 127.95, 127.41, 126.51, 122.68, 81.12, 80.63, 71.32, 70.62, 55.30, 42.48, 38.85, 29.39, 28.13, 28.10, 27.99, 26.79.

MS (ESI) C45H51NO7S: m/z 750 $[M]^+$.

Spectra

<u>[^{19}F]FRPG</u>

^{1}H NMR

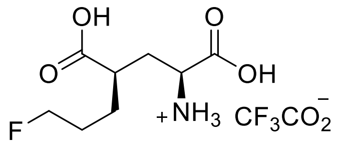

[^{19}F]FRPG

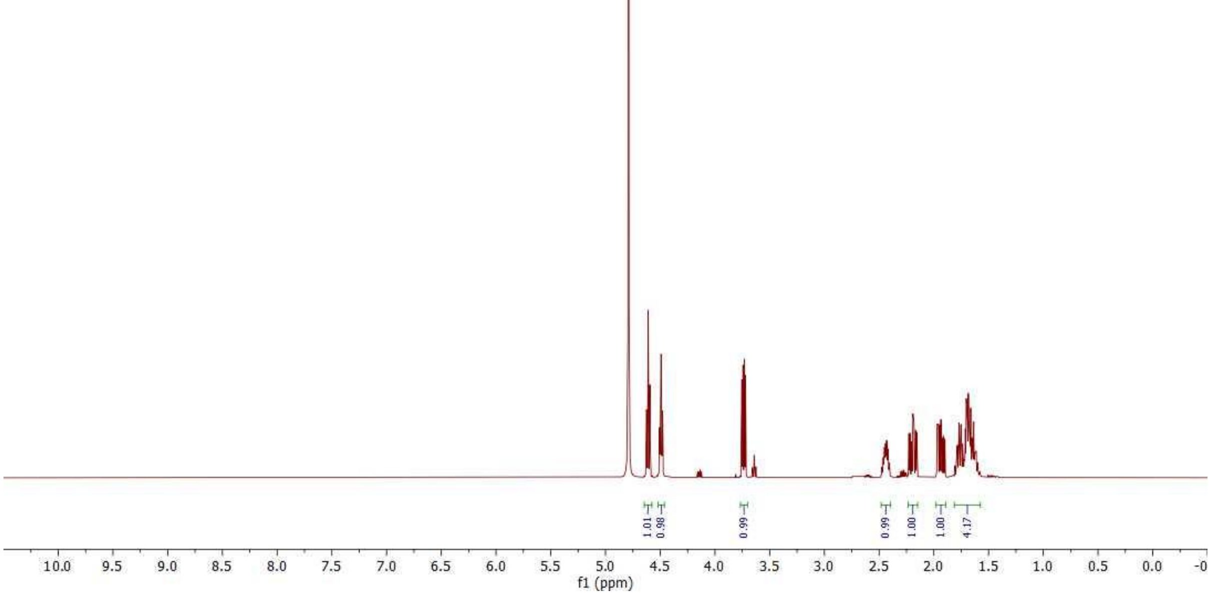

^{13}C NMR

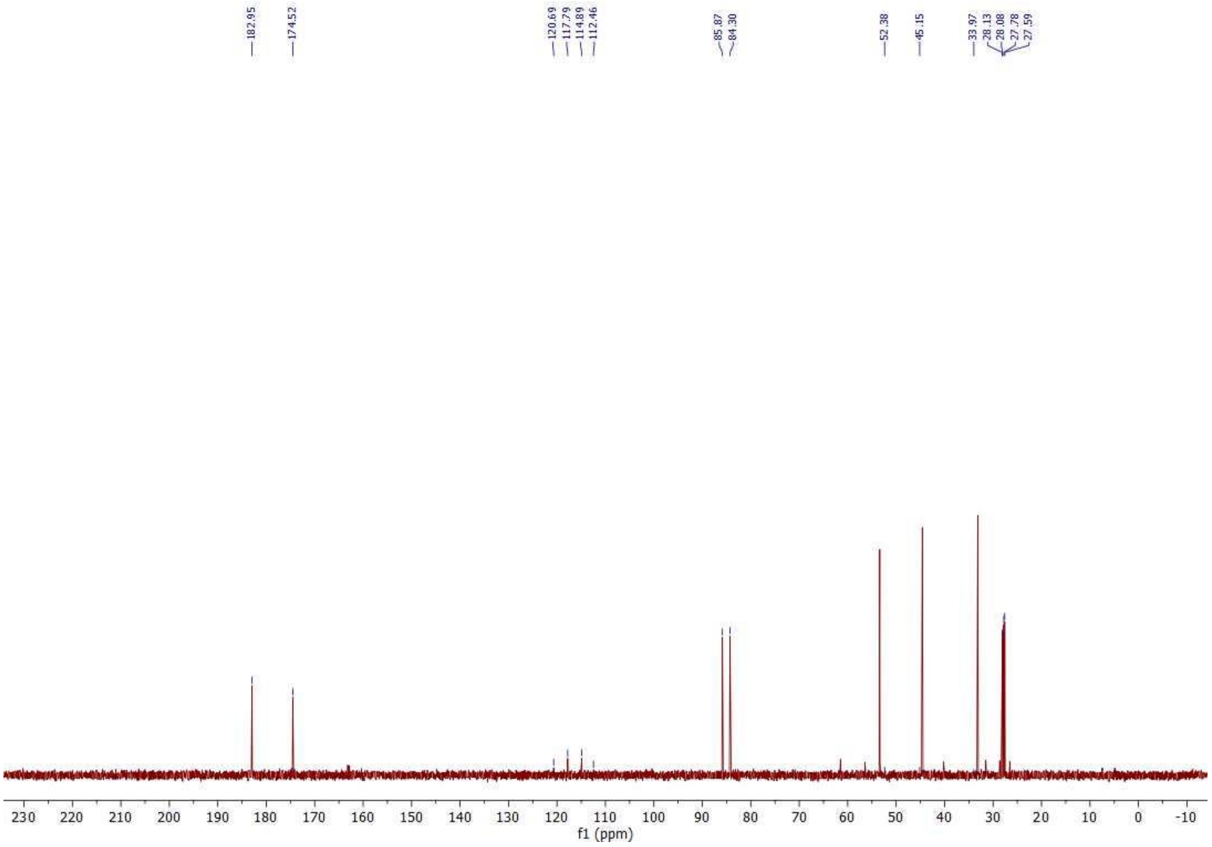

182.95
174.52
120.69
117.79
114.89
112.46
85.87
84.30
52.38
45.15
33.97
28.13
28.08
27.78
27.59
230 220 210 200 190 180 170 160 150 140 130 120 110 100 90 80 70 60 50 40 30 20 10 0 -10
f1 (ppm)

^{19}F NMR

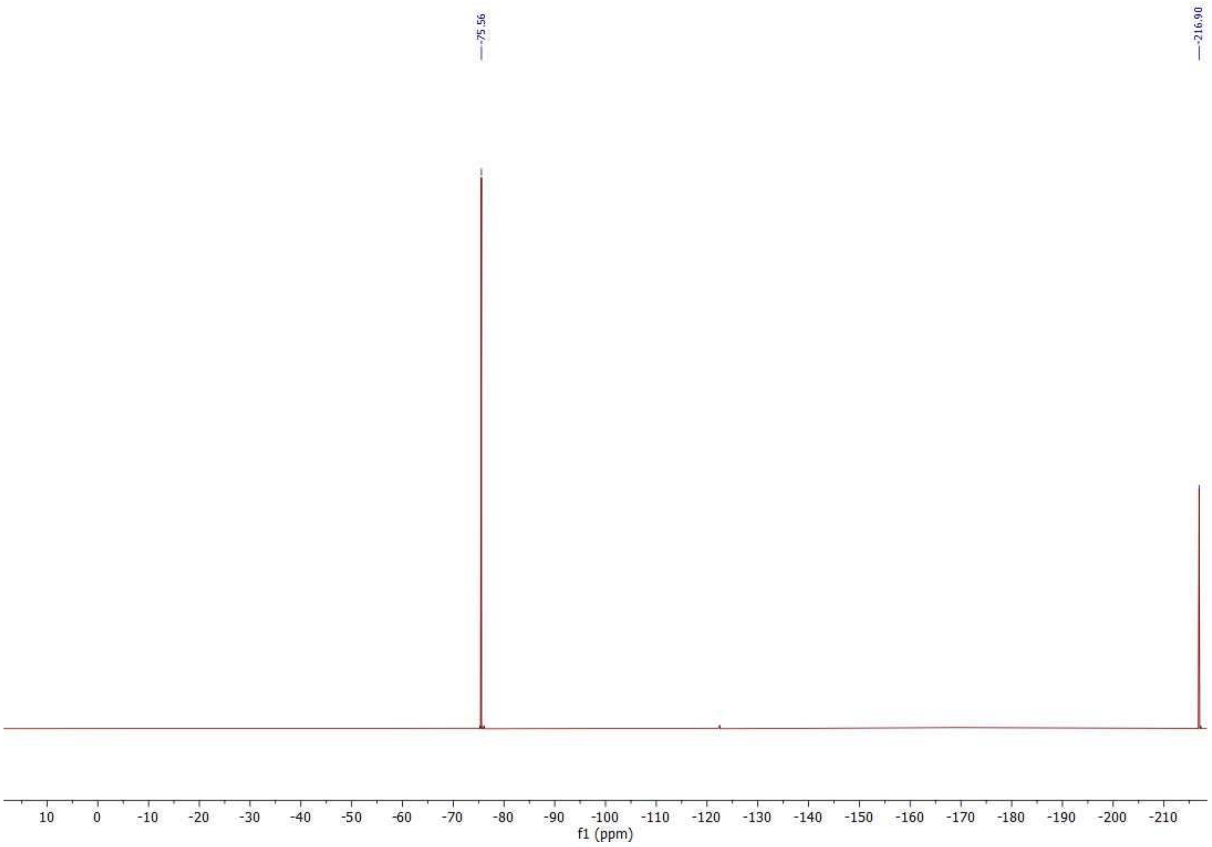
-75.56
-216.90
10
0
-10
-20
-30
-40
-50
-60
-70
-80
-90
-100
-110
-120
-130
-140
-150
-160
-170
-180
-190
-200
-210
f1 (ppm)

[19F]FSPG

^{1}H NMR

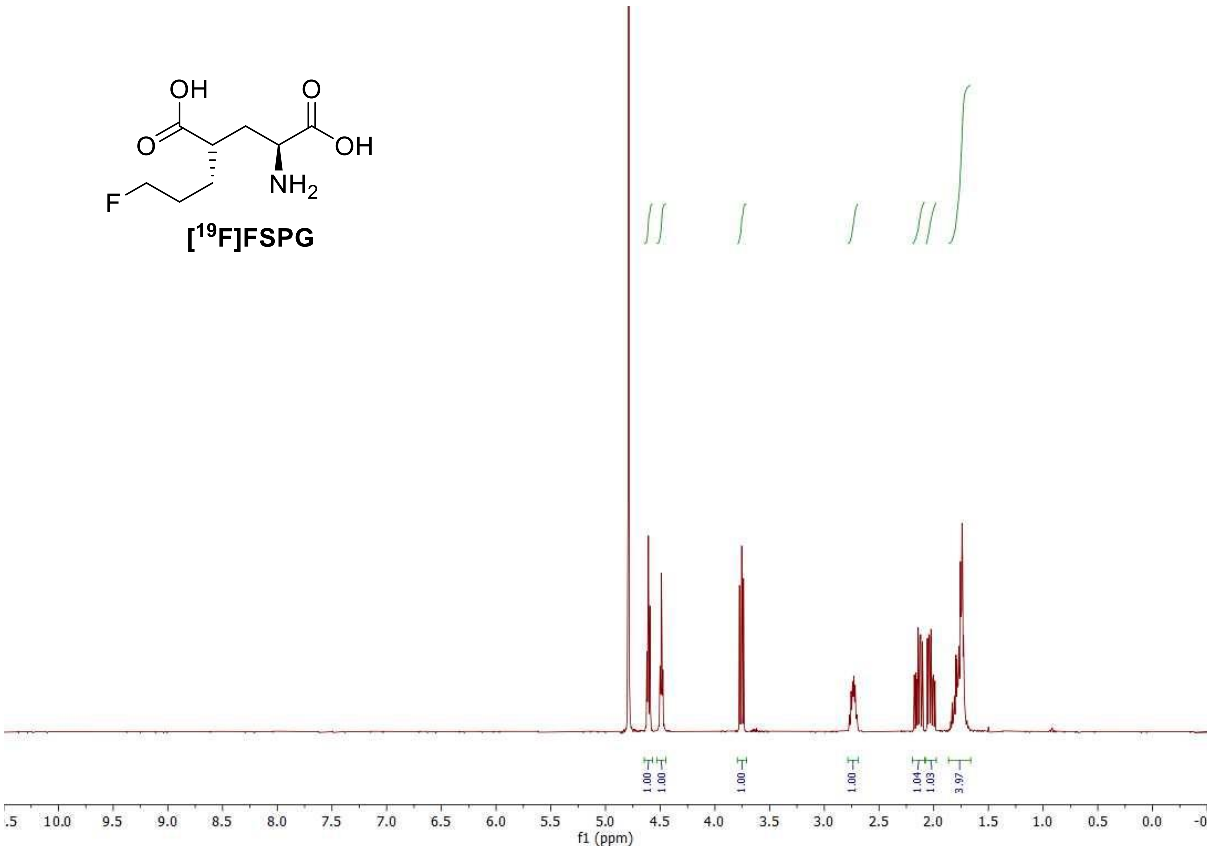

^{13}C NMR

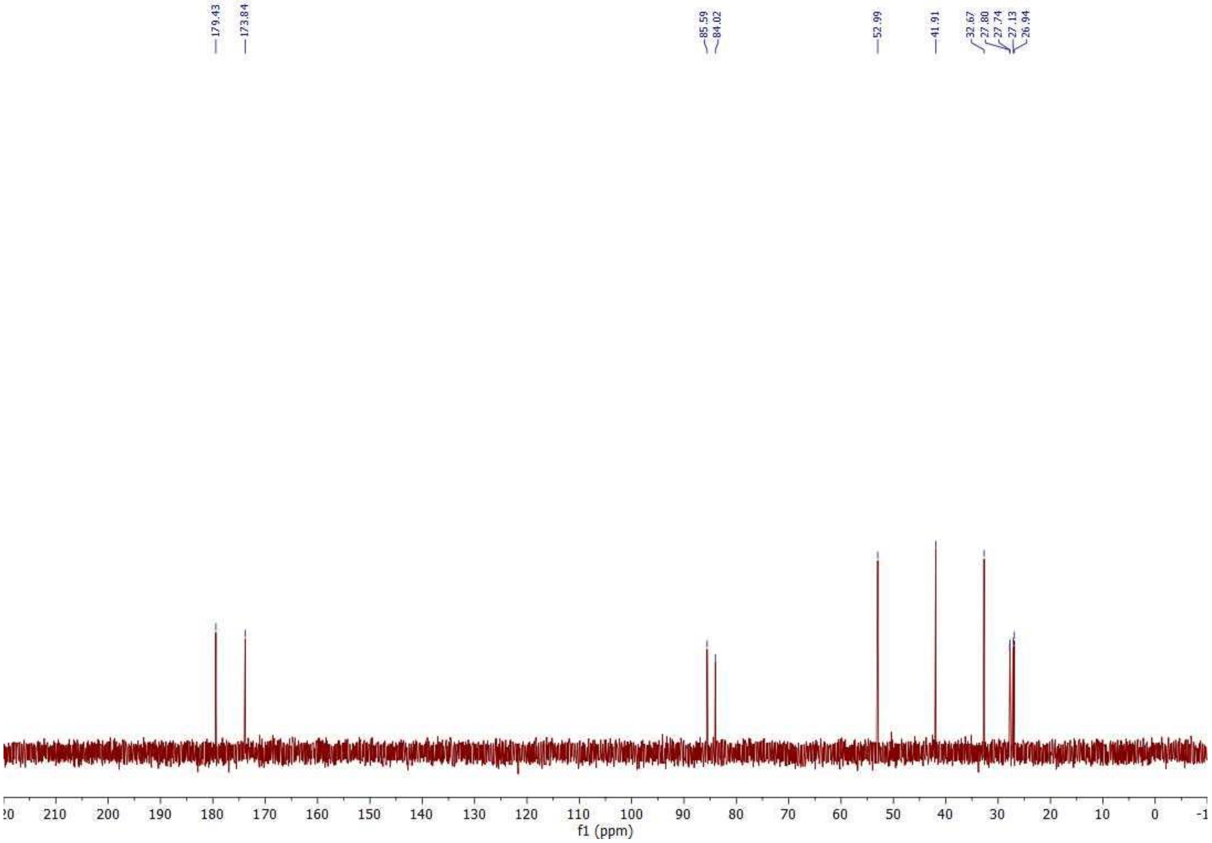
179.43
173.84
85.59
84.02
52.99
41.91
32.67
27.80
27.74
27.13
26.94
20 210 200 190 180 170 160 150 140 130 120 110 100 90 80 70 60 50 40 30 20 10 0 -1
f1 (ppm)

^{19}F NMR

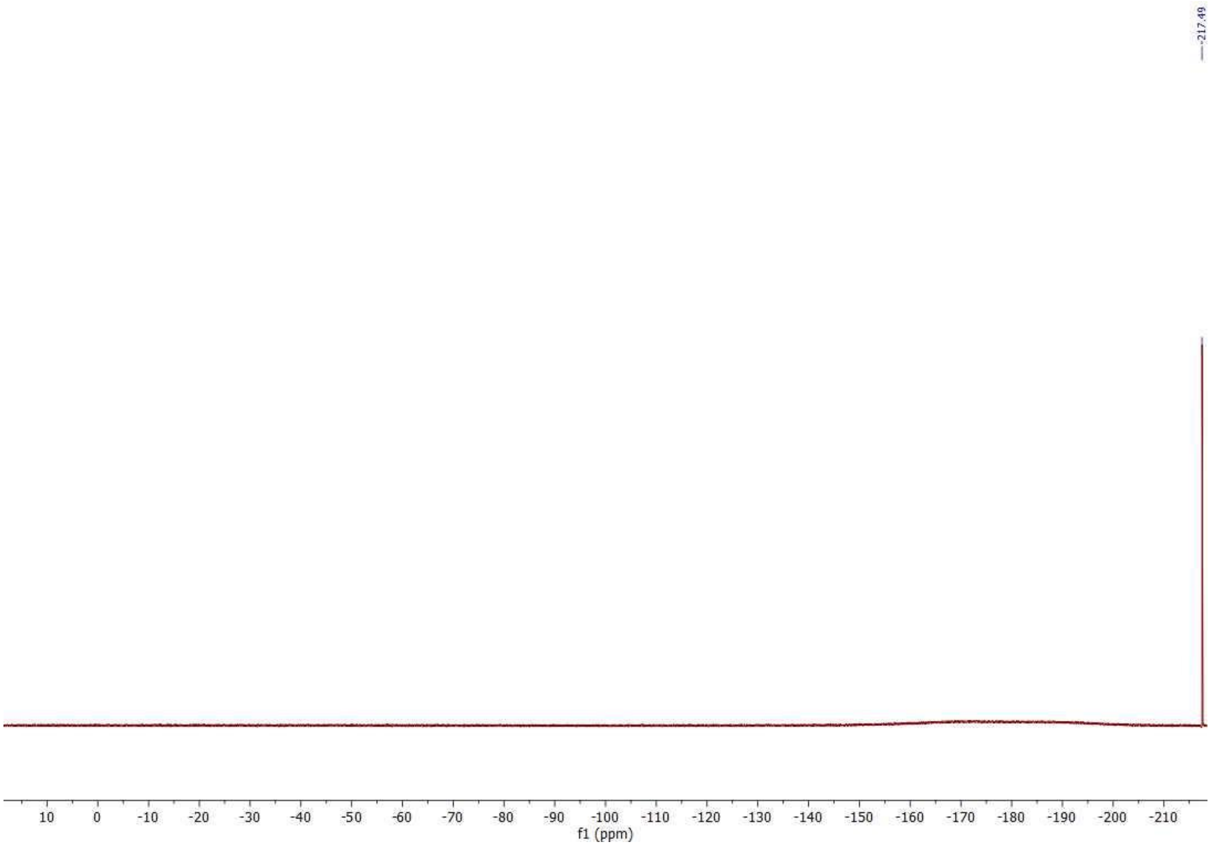

-217.49
10 0 -10 -20 -30 -40 -50 -60 -70 -80 -90 -100 -110 -120 -130 -140 -150 -160 -170 -180 -190 -200 -210
f1 (ppm)

[18F]FRPG Precursor ((2*S*,4*R*)-**12**)

^{1}H NMR

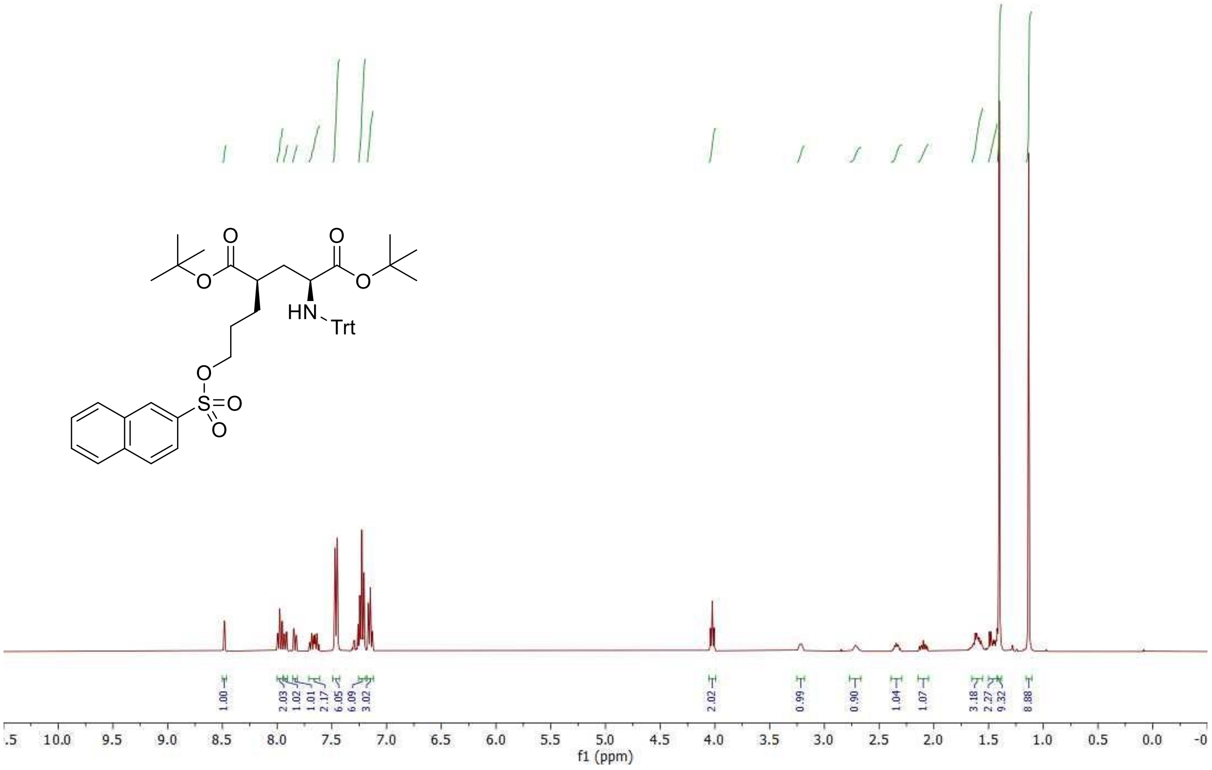

^{13}C NMR

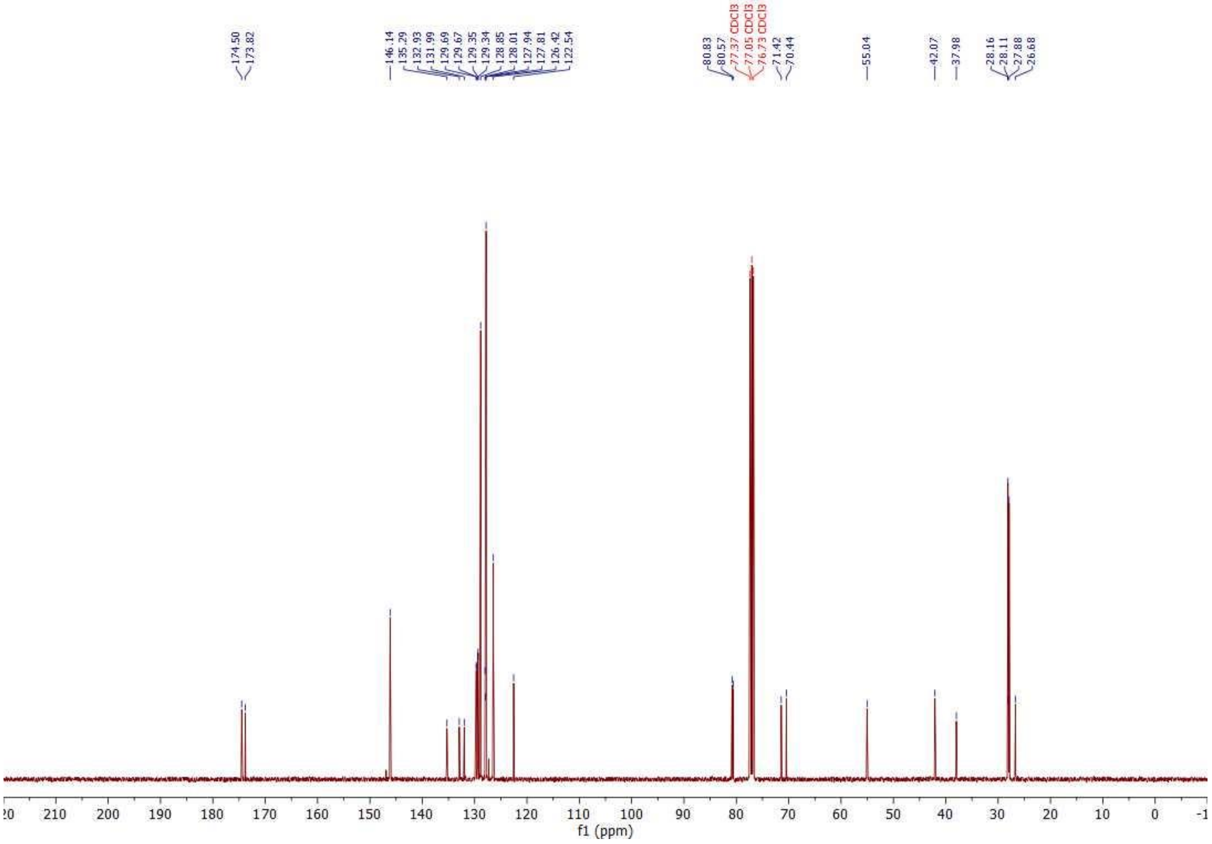
174.50
173.82
146.14
135.29
132.93
131.99
129.69
129.67
129.35
129.34
128.85
128.01
127.94
127.81
126.42
122.54
80.83
80.57
77.37 CDCl3
77.05 CDCl3
76.73 CDCl3
71.42
70.44
55.04
42.07
37.98
28.16
28.11
27.88
26.68
f1 (ppm)

[18F]FSPG Precursor ((2*S*,4*S*)-**12**)

^{1}H NMR

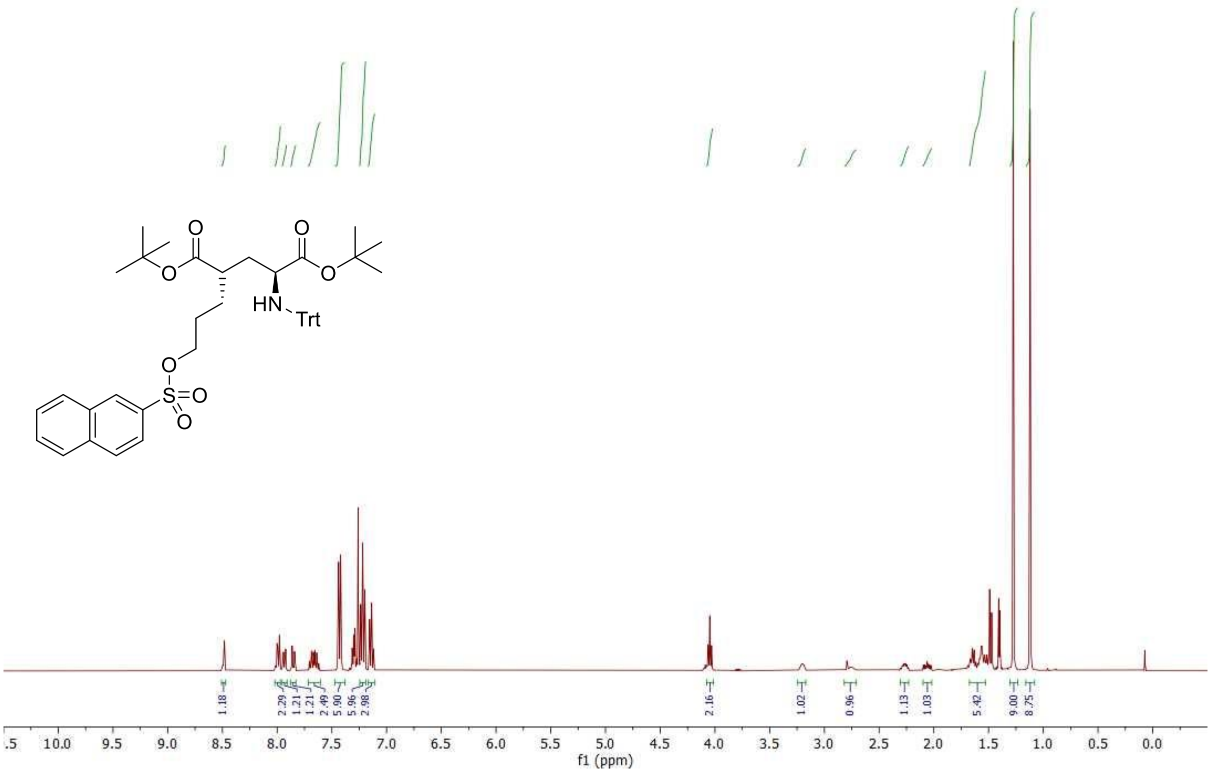

^{13}C NMR

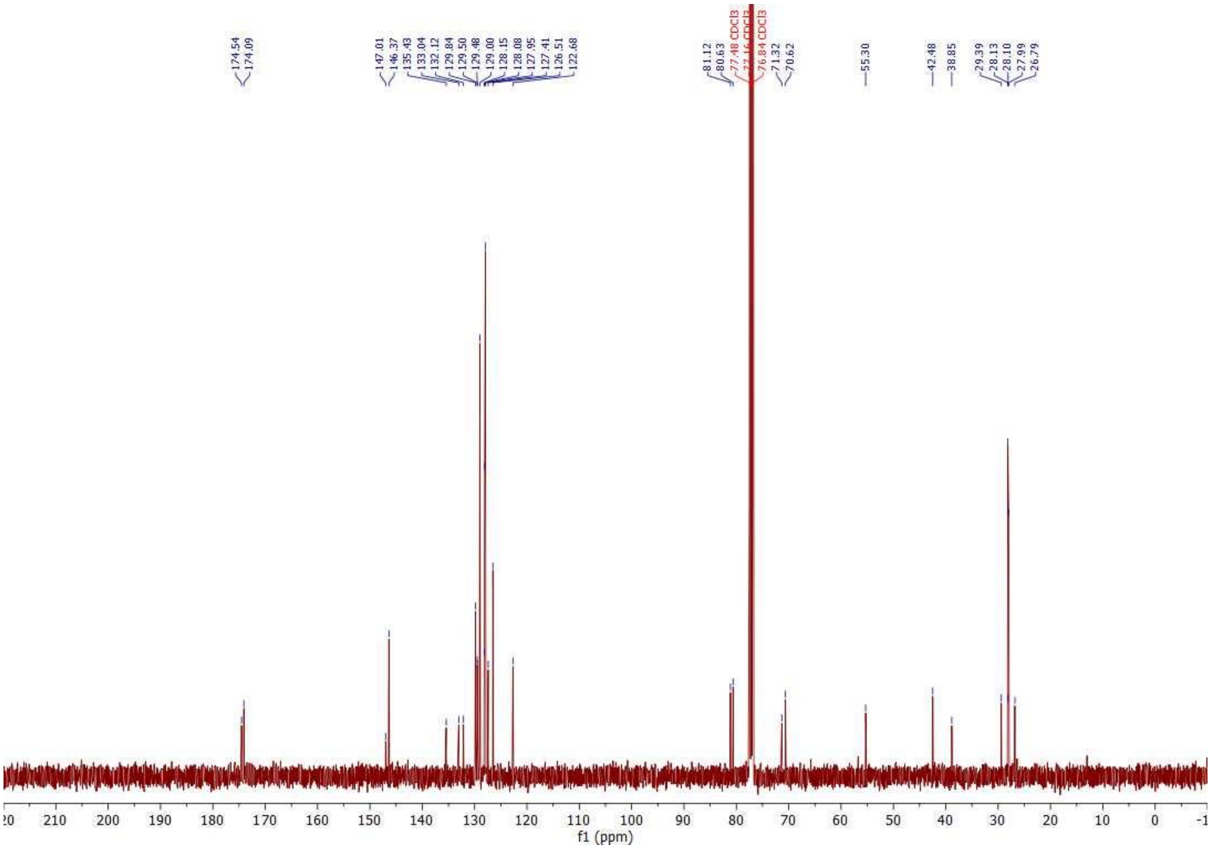

References

1. Shih KT, Huang YY, Yang CY, Cheng MF, Tien YW, Shiue CY, et al. Synthesis and analysis of 4-(3-fluoropropyl)-glutamic acid stereoisomers to determine the stereochemical purity of (4S)-4-(3-[18F]fluoropropyl)-L-glutamic acid ([18F]FSPG) for clinical use. PLoS One. 2020; 15: e0243831.

2. Dinkelborg L, Schmitt-Willich H, Graham K, Koglin N, Berndt M, Friebe M, Muller A. US2014/0227191 A1, 2014.

3. Berndt M, Schmitt-Willich H, Friebe M, Graham K, Brumby T, Hultsch C, et al. WO2011/060887 A2, 2011.

4. Han G, Tamaki M, Hruby VJ. Fast, efficient and selective deprotection of tert-butoxycarbonyl (Boc) group using HCl/dioxane (4M). J. Peptide Res. 2001; 58: 338–341.

5. Hultsch C, Harre M, Novak F, Berndt M, Friebe M, Schmitt-Willich H, et al. WO2012/150204 A1, 2012.
